# Influence and Optimization of Landscape Elements on Outdoor Thermal Comfort in University Plazas in Severely Cold Regions

**DOI:** 10.3390/plants14142228

**Published:** 2025-07-18

**Authors:** Zhiyi Tao, Guoqiang Xu, Guo Li, Xiaochen Zhao, Zhaokui Gao, Xin Shen

**Affiliations:** 1 Architecture College, Inner Mongolia University of Technology, Hohhot 010051, China; 20211800609@imut.edu.cn (Z.T.); 20211100430@imut.edu.cn (G.L.); ayzhaoxiaochen@126.com (X.Z.); 13033424364@163.com (Z.G.); 20231800692@imut.edu.cn (X.S.); 2 The Key Laboratory of Grassland Habitat System and Low-Carbon Construction Technology, Hohhot 010051, China; 3 Key Laboratory of Green Building at Universities of Inner Mongolia Autonomous Region, Hohhot 010051, China

**Keywords:** severely cold regions, landscape elements, outdoor thermal comfort, orthogonal experiment, numerical simulation, climate-responsive design, nature-based solutions

## Abstract

Universities in severely cold regions face the dual challenge of adapting to seasonal climate variations while enhancing outdoor thermal comfort in outdoor leisure plazas. This study takes a university in Hohhot as a case study. Through field investigations conducted in summer and winter, thermal benchmarks were established. Based on this, an orthogonal experimental design was developed considering greenery layout, plant types, and surface albedo. ENVI-met was used to simulate and analyze the seasonal regulatory effects of landscape elements on the microclimate. The results show that: (1) the lower limit of the neutral PET range in Hohhot in winter is −11.3 °C, and the upper limit in summer is 31.3 °C; (2) the seasonal contribution of landscape elements to PET ranks as follows: plant types > greenery layout > surface albedo; and (3) the proposed optimization plan achieved a weighted increase of 6.0% in the proportion of activity area within the neutral PET range in both summer and winter. This study is the first to construct outdoor thermal sensation categories for both summer and winter in Hohhot and to establish a thermal comfort optimization evaluation mechanism that considers both diurnal and seasonal weightings. It systematically reveals the comprehensive regulatory effects of landscape elements on the thermal environment in severely cold regions and provides a nature-based solution for the climate-responsive design of campus plazas in such areas.

## 1. Introduction

With the intensification of global warming and the acceleration of urbanization, the thermal environment in urban outdoor spaces has been continuously deteriorating [1]. As an important component of urban open space, university outdoor leisure plazas in severely cold regions face the dual challenges of summer overheating and severe winter cold. Defining their outdoor thermal benchmarks for both summer and winter and revealing the seasonal regulatory effects of landscape elements on the microclimate are critical for adapting to seasonal climate variations and enhancing users’ thermal comfort. Therefore, based on the established thermal benchmarks for university outdoor leisure plazas in severely cold regions [2], this study aims to provide an effective nature-based solution for the climate-responsive design of urban plazas in such climatic zones by optimizing greenery layout, plant types, and surface albedo [3].

The climatic zones, seasonal coverage, evaluation indices, and research focus of studies on outdoor thermal comfort in university settings are essential for clarifying the innovation of this study. The existing literature shows that only a few studies have focused on universities in severely cold regions [4], while most have concentrated on cold regions [5,6,7], hot summer and cold winter regions [8,9,10,11,12,13,14], and hot summer and warm winter regions [15,16,17,18,19,20,21,22]. Among these, only a limited number of studies cover both summer and winter seasons [4,5,8,10,22,23]. Furthermore, the vast majority of studies use Physiologically Equivalent Temperature (PET) as the main index for evaluating outdoor thermal comfort [4,5,6,7,9,12,13,14,15,16,17,19,20,21,22,23]. Lastly, only a few have addressed the outdoor thermal perception and thermal preferences of respondents [4,21,23].

The influence of landscape elements on outdoor thermal comfort varies greatly across climate zones, study locations, seasonal coverage, and research content. Compared to architectural elements, landscape features have greater potential to enhance outdoor thermal comfort in both summer and winter. Although building morphology parameters, such as the height-to-width ratio and orientation, have a decisive impact on microclimate conditions [24], their adjustability in built environments is limited. Additionally, water features are expensive to maintain in arid regions [3].

Existing studies have confirmed that green space layout, plant species, and ground surface albedo significantly influence outdoor thermal comfort. Larger green areas with more complex shapes tend to have stronger cooling effects. For example, Cao et al. found that irregular, belt-shaped parks exhibited stronger cooling effects [25]. Li et al. pointed out that block-shaped, clustered, and large-scale urban green spaces help significantly reduce surrounding thermal loads [26]. Zhou et al. further emphasized that green spaces with a high Land Shape Index (LSI) are more effective at enhancing shade and promoting thermal interaction between green areas and built environments [27].

In addition, plants with a high Leaf Area Index (LAI) exhibit stronger thermal regulation capabilities. Research shows that properly configured trees can reduce Predicted Mean Vote (PMV) by up to 1.75 in summer and increase it by 0.50 during the coldest winter periods [28]. Dong et al. also found that Acer species provide the highest cooling intensity, while Pinus species provide the least [29].

Moreover, the albedo of ground surface materials significantly affects surface temperatures and pedestrian thermal sensation. High-albedo materials can effectively reduce surface temperatures [30]. Santamouris et al. found that high-reflectance pavements can lower peak summer air temperature by 1.9 °C and surface temperature by 12 °C [31], but excessive surface reflectivity may reduce thermal comfort at pedestrian height [32]. On the other hand, low-albedo materials absorb more heat, potentially increasing pedestrian discomfort [33].

Previous studies have shown that only a few have been conducted in cold or severely cold regions [29,34,35,36]. While most studies have covered both summer and winter seasons [25,28,34,36,37], only one study has been conducted on a university campus [28]. Furthermore, most studies have focused on the impact of a single landscape element on outdoor thermal comfort [37]. Although the number of studies on outdoor thermal comfort in severely cold regions is limited, this climate zone accounts for about one-quarter of China’s land area [38], and related research has mainly concentrated on typical cities in northeast China, such as Harbin, with relatively less focus on the northwest region.

In summary, research on outdoor thermal comfort in university leisure plazas in severely cold regions, particularly in northwest China, remains insufficient. There is a lack of comprehensive studies that assess the combined impact of various landscape elements on outdoor thermal comfort across both summer and winter seasons. Therefore, this study focuses on an outdoor leisure plaza at a university in Hohhot, located in northwest China. By combining field surveys and numerical simulations conducted in both summer and winter, and establishing the thermal benchmarks for both seasons, this study systematically investigates the impact of various landscape elements, including greenery layout, plant species, and surface albedo, on outdoor thermal comfort. The aim is to fill the research gap in this field and propose nature-based solutions to optimize landscape elements for improving outdoor thermal comfort.

Based on the identified research gaps, this study takes the outdoor leisure plaza of a university in Hohhot as the research subject. After evaluating the thermal comfort conditions in both summer and winter, it further explores the influence of greenery layout, plant types, and surface albedo on outdoor thermal comfort.

The research objectives are as follows:

(1) To determine the outdoor thermal benchmarks for plaza users during summer and winter;
(2) To quantify the impact, significance, and contribution of different landscape elements on thermal comfort;
(3) To identify the optimal design scheme for thermal comfort across both summer and winter seasons;
(4) To propose nature-based solutions tailored for severely cold regions.

The findings aim to provide a theoretical foundation for the landscape design of outdoor spaces in universities located in cold regions, with the goal of creating outdoor activity spaces that balance climate adaptability with health and comfort.

## 2. Methodology

### 2.1. Study Site and Field Measurements

#### 2.1.1. Study Site

**Figure 1:**
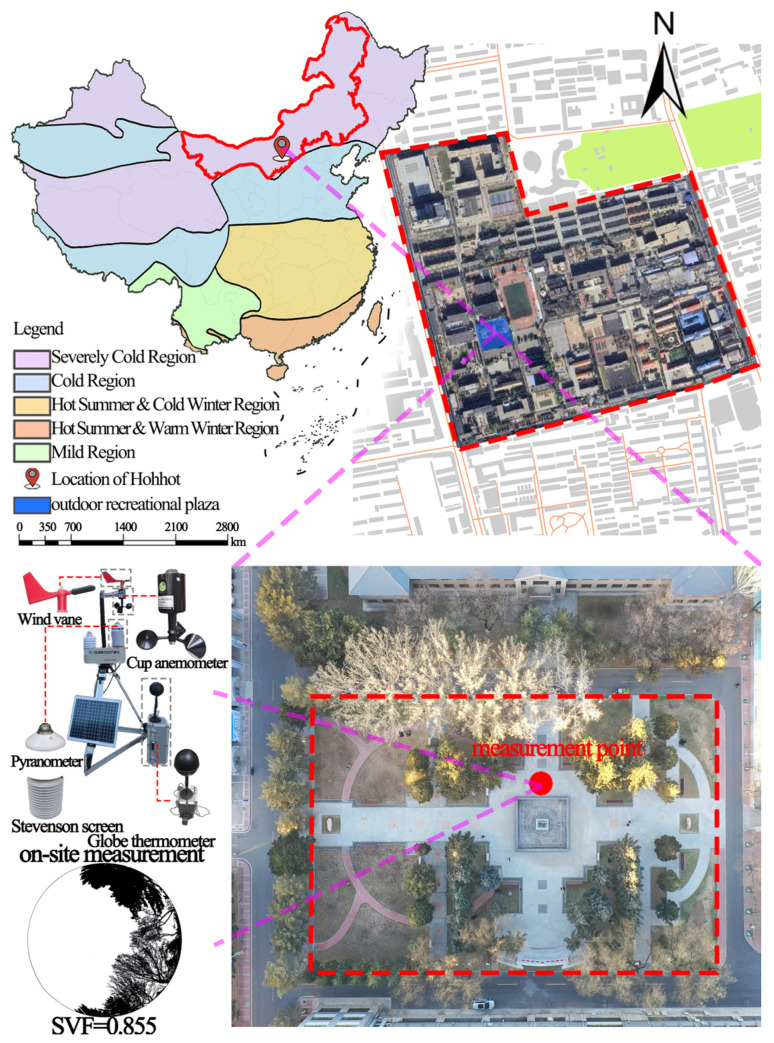
Research object.

Hohhot (110°46′–112°10′ E, 40°51′–41°8′ N), located in northwest China, has a distinct four-season climate. Winters are long and severely cold, while summers are short and hot. Spring and autumn serve as transitional periods, often characterized by large diurnal temperature variations and frequent temperature fluctuations [39]. In this study, an outdoor leisure plaza at a university in Hohhot was selected as the research site, as shown in Figure 1.

The square has a rectangular layout, diverse plant types, and various paving forms, making it quite representative. Additionally, based on the crowd gathering characteristics, sky cover, and landscape elements within the square, a monitoring point was set up on the north side of the fountain to collect outdoor thermal environment parameters for subsequent thermal comfort assessment.

#### 2.1.2. Survey Dates

**Figure 2:**
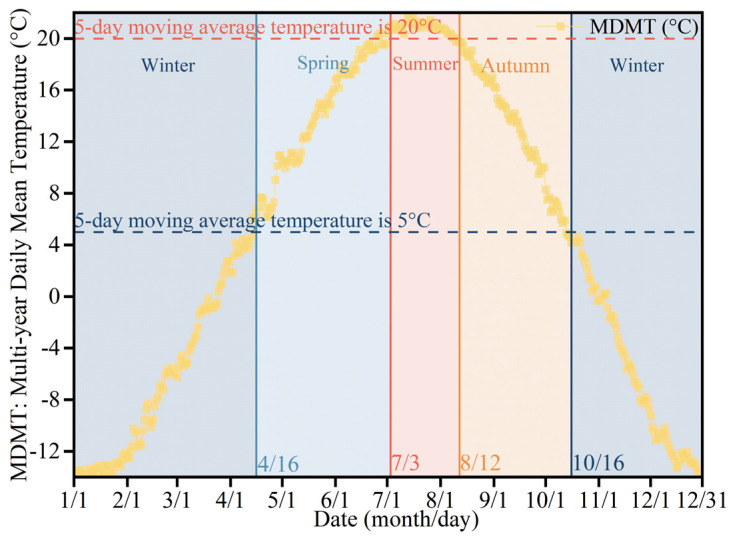
Seasonal division in Hohhot.

This study used the 5-day moving average temperature method to analyze the daily mean temperature data from Hohhot over the past 30 years (1994–2023) [40], and then determined the seasonal divisions [41], as shown in Figure 2.

Considering the number of outdoor activities on campus, June and December were selected as the representative meteorological months for investigating outdoor thermal comfort in summer and winter, respectively.

This study selected hourly air temperature, relative humidity, wind speed, and horizontal solar radiation as the main meteorological parameters for June and December [42], with weights assigned in the ratio of 1:1:1:3 [43,44]. By calculating the cumulative weighted Mean Absolute Percentage Error (MAPE), 29 June and 7 December 2024 were identified as the typical meteorological days (TMDs) for summer and winter, respectively.

The specific steps for determining the typical meteorological days (TMDs) based on the MAPE are as follows:

(1) Obtain the hourly values of air temperature, relative humidity, wind speed, and horizontal solar radiation for each day, and calculate the hourly mean values of each parameter for the month;
(2) Compute the MAPE of each parameter by comparing the hourly values of each day against the monthly hourly averages;
(3) Apply a weighted aggregation of the MAPE values using the weight ratio 1:1:1:3 across the four parameters. The day with the smallest cumulative weighted MAPE is selected as the typical meteorological day.

The calculation is expressed as follows:

(1) $$MAPE=\frac{1}{n}\sum_{i=1}^{n}\frac{\left|X_{day,i}-X_{month,i}\right|}{\left|X_{month,i}\right|}$$

(2) $${WS}_{d}=\sum_{i=1}^{4}W_{i}{MAPE}_{i}$$

In these formulas:

$X_{day,i}$ denotes the hourly value of a meteorological parameter on a given day at hour i;
$X_{month,i}$ represents the corresponding hourly average of that parameter for the month;
n represents the number of selected hours per day;
${WS}_{d}$ refers to the cumulative weighted Mean Absolute Percentage Error for a given TMD;
$W_{i}$ is the weight assigned to the i-th meteorological parameter;
${MAPE}_{i}$ is the MAPE of the i-th parameter on the candidate day.

#### 2.1.3. Field Measurements

In this study, a portable meteorological station (PC-6) with radiation shielding was used to measure air temperature (Ta), relative humidity (RH), and wind speed (Va). A JTR-04 globe thermometer with a diameter of 150 mm was employed to record globe temperature (Tg). All instruments met the measurement range and accuracy requirements specified in ISO 7726 (1998) [45]. Detailed information about the equipment, including measurement ranges and accuracies, is provided in Table 1.

The mean radiant temperature (Tmrt) was calculated using the following equation:

(3) $$T_{mrt}={\left[{\left(T_{g}+273.15\right)}^{4}+\frac{\left(1.1\times 10^{8}\times {Va}^{0.6})(Tg-Ta\right)}{\varepsilon D^{0.4}}\right]}^{1/4}$$

In this formula:

$T_{g}$ is the globe temperature measured by the globe thermometer (°C);
$v_{a}$ is the wind speed measured by the portable meteorological station (m/s);
$T_{a}$ is the air temperature measured by the portable meteorological station (°C);
D is the diameter of the globe thermometer (m, the standard black globe with a diameter of 0.15 m was used for measurements in this study);
ε is the emissivity of the globe surface (taken as 0.95 in this study).

To ensure the representativeness of the measured meteorological data, outdoor thermal environment measurements were conducted on the selected typical meteorological days. These dates were carefully chosen to avoid adverse weather conditions, such as wind, snow, rain, fog, and dust storms, and to exclude the influence of organized activities that might affect respondents’ actual thermal sensations. Since measurements were taken on weekends, students were mainly engaged in spontaneous outdoor activities, which helped to more accurately reflect their true thermal perceptions. The measurements were carried out from 09:00 to 18:00 at 1 min intervals. All instruments were placed at a height of 1.5 m. During the administration of outdoor thermal comfort questionnaires, the distance between the monitoring instruments and the respondents was maintained between 3 and 9 m to ensure that the recorded environmental data accurately reflected participants’ actual thermal sensations [46,47].

### 2.2. Subjective Questionnaire Survey

#### 2.2.1. Questionnaire Structure and Reliability Testing

To ensure the reliability of the survey results, respondents were randomly selected from individuals participating in outdoor activities within the plaza during the specified observation periods. The questionnaire consisted of three main parts, detailed in Appendix A.1 and Appendix A.2.

Part 1 collected respondents’ physiological characteristics, including gender, age, height, weight, clothing insulation level [45], survey time period, current location, and type of activity.

Part 2 focused on individual background characteristics, such as duration of residence in Hohhot, main outdoor activity times during the day, type and setting of activity in the 15 min prior to the survey, activity purpose, frequency, duration, and primary location.

Part 3 gathered respondents’ subjective thermal perceptions, including thermal sensation, thermal comfort, thermal acceptability, and preferences for thermal environmental parameters. The questionnaire employed a 7-point thermal sensation scale, a 4-point thermal comfort scale, a 4-point thermal acceptability scale, and a 3-point thermal preference scale to quantify subjective responses.

The minimum required sample size for the questionnaire was estimated using the formula proposed by Du et al. [48], indicating that at least 97 valid responses were needed for both summer and winter seasons in the university outdoor leisure plaza.

(4) $$N=\frac{{\left(Z_{1-\alpha /2}\right)}^{2}\sigma^{2}}{E^{2}}$$

In this formula:

α represents the probability of a Type I error, which is generally set at α = 0.05;
$Z_{0.975}$ is the Z-score corresponding to a 95% confidence level, Z_0.975_ = 1.96;
σ denotes the population standard deviation, σ = 1.5 [49];
E represents the acceptable margin of error, set at 5% of the thermal sensation scale length (E = 0.3).

To ensure the validity of the questionnaire responses, this study applied the criterion |TSV + TPV| ≥ 3, where a value greater than or equal to 3 indicates contradictory answers and is treated as an invalid questionnaire [50].

After validity screening, 135 valid questionnaires were obtained for summer and 204 for winter, both meeting the sample size requirements for outdoor thermal comfort surveys.

Furthermore, reliability analyses were conducted separately for thermal sensation, thermal comfort, and thermal acceptability in both summer and winter. The Cronbach’s α coefficient was calculated using the following formula [51]:

(5) $$\alpha =\frac{K}{1-K}\times (1-\frac{\sum_{1}^{K}S_{i}^{2}}{S_{Sum}^{2}})$$

In this formula:

K represents the number of items in the questionnaire;
$S_{i}^{2}$ is the variance of the i-th item;
$S_{sum}^{2}$ is the total variance of all items.

The Cronbach’s α values for the outdoor thermal comfort surveys in summer and winter were 0.719 and 0.787, respectively. Since both values fall within the range of 0.7 to 0.8, the questionnaire is considered to have good reliability and meets the reliability requirements.

#### 2.2.2. Outdoor Thermal Comfort Index

This study selected Physiological Equivalent Temperature (PET) as the thermal comfort evaluation index. PET is derived from the Munich Energy-balance Model for Individuals (MEMI) [52] and is used to describe the air temperature at which, under a specific outdoor environment, the skin and core temperatures of the human body are the same as those in a typical indoor environment (without wind and solar radiation) when the body is in thermal equilibrium [53]. PET is expressed in degrees Celsius (°C) and has been widely used in outdoor thermal comfort studies across different climate zones [54].

In this study, PET values were batch-calculated using the open-source software RayMan Pro. The calculation incorporated meteorological parameters obtained from field measurements (air temperature, relative humidity, wind speed, and mean radiant temperature), as well as physiological data collected from the questionnaires (such as age, gender, height, weight, clothing insulation, and metabolic rate) [55].

### 2.3. Experimental Design

#### 2.3.1. Configuration of Landscape Elements

This study considers vegetation layout, plant type, and surface albedo as the three factors influencing outdoor thermal comfort, along with their respective levels, as shown in Table 2.

(1) Greening Layout (A):

Under the premise of ensuring that the total green area for each experimental scheme is 2592 m^2^, the layout was designed based on the site’s existing eight green plots. Each plot was set to an area of 324 m^2^, with dimensions of 18 m × 18 m. To maintain proportional relationships between the dimensions of dot-shaped, strip-shaped, and block-shaped green areas—and to facilitate pedestrian guidance and spatial separation—the dimensions were defined as follows: dot-shaped green areas at 3 m × 3 m and strip-shaped green areas at 3 m × 6 m.

The specific dimensions and quantities are as follows [56]: dotted-shaped green spaces (3 m × 3 m, 288 units), strip-type green spaces (3 m × 6 m, oriented both N–S and W–E, 144 units in total), and block-type green spaces (18 m × 18 m, 8 units).

(2) Plant Type (B):

Under the condition that each experimental scheme contains 144 plants, privet—being the only shrub species present on-site—was included in the planting scheme. Among trees with a height of 15 m or less, Chinese plum (*Prunus triloba*) and Chinese pine (*Pinus tabuliformis*) each accounted for more than 25% of the total plant count and were, therefore, selected as representative tree species. For trees 20 m or taller, black poplar (*Populus nigra*) had the highest proportion and was also incorporated into the plaza’s greening plan. The parameters of these plant species are shown in Table 3.

(3) Surface Albedo (C):

Surface materials with albedos of 0.2, 0.3, 0.4, and 0.5 were adopted to ensure thermal comfort and visual acceptability. Materials with albedos below 0.2 (e.g., asphalt) were avoided due to heat accumulation, as were highly reflective materials exceeding 0.6 (e.g., white gravel), which may cause visual discomfort [33].

#### 2.3.2. Orthogonal Experimental Design

The orthogonal experimental design is a statistical method that utilizes orthogonal arrays to select representative combinations of factors and levels [58]. It enables the efficient evaluation of the main effects and interactions among multiple factors on the experimental outcomes, thereby reducing the number of required tests and significantly improving experimental efficiency [59]. This method has been widely applied in outdoor thermal environment research [60,61,62].

In this study, an L16(4^5^) orthogonal array was employed. The first three columns were assigned to the three experimental factors: greening layout (A), plant type (B), and surface albedo (C). The remaining two columns were left blank for error analysis. A total of 16 experimental schemes were generated (see Table A1 in Appendix A.3 for details). The balanced distribution of the array was used to determine the order of influence of the main effects. Furthermore, analysis of variance (ANOVA) with an F-test at a significance level of α = 0.05 was conducted to quantify the significance and contribution rate of each factor.

#### 2.3.3. ENVI-met Boundary Conditions

ENVI-met is a three-dimensional urban microclimate simulation software based on computational fluid dynamics and thermodynamics and is widely used to assess the impact of vegetation on urban microclimates [63]. In this study, ENVI-met V5.7.2 was employed to simulate the thermal environment in a university outdoor recreational plaza during both summer and winter, aiming to evaluate the influence of landscape elements on outdoor thermal comfort.

To ensure the validity of the simulation results, partial correlation analysis was conducted. The results revealed that the air temperature (Ta) and mean radiant temperature (Tmrt) were significantly positively correlated with PET, while wind speed (Va) was significantly negatively correlated, as shown in Table 4.

Considering that current ENVI-met simulation validation studies prioritize Ta as the primary parameter and Tmrt as a secondary parameter [64], and that studies involving wind speed and direction are relatively few due to their hourly dynamic variability [65], this study selected Ta and Tmrt as the main parameters for model validation.

The boundary conditions for the ENVI-met model are presented in Table 5. The simulation was initialized at 07:00 and run for a duration of 11 h to exclude the initial 2 h model spin-up period [35].The model domain consisted of 241 × 252 × 17 grids, with a spatial resolution satisfying the domain requirements in both horizontal [66] and vertical directions [67]. The horizontal grid size was set to 1 m × 1 m, and the initial vertical grid height was 1 m, with a vertical stretching factor of 20% applied above 2 m [68]. The model’s actual orientation was offset by 13.54° from true north.

The calculation of specific humidity at 2500 m was conducted following the procedure described by Forouzandeh [67]. The wind speed and direction at 10 m height for both summer and winter simulations were based on observational data from a fixed meteorological station located on the campus at a height of 10 m above ground level. The value for the surface microscale roughness length was adopted from the study by Stull [69], while the cloud cover data were obtained from publicly available NASA sources [70].

Additionally, the physiological characteristics of the respondents, as collected through the questionnaire survey, were input into the BioMet module of ENVI-met to compute PET values for the plaza in summer and winter. The physiological characteristics of the respondents are summarized in Table 6.

#### 2.3.4. Model Accuracy Validation

The accuracy of the ENVI-met model was evaluated by comparing the simulated and measured values of air temperature (Ta) and the mean radiant temperature (Tmrt) at a height of 1.5 m on typical meteorological days in both summer and winter. The model’s performance was assessed using the root mean square error (RMSE) and mean absolute error (MAE) [2].

(6) $$RMSE=\sqrt[{2}]{\sum_{i=1}^{n}\frac{{\left(P_{i}-O_{i}\right)}^{2}}{n}}$$

(7) $$MAE=\frac{\sum_{i=1}^{n}\left|P_{i}-O_{i}\right|}{n}$$

In these formulas:

- $P_{i}$: predicted value;
- $O_{i}$: observed value;
- n: number of cases;
- RMSE: reflects the overall deviation of the predictions;
- MAE: directly reflects the average magnitude of the prediction error.

As shown in Table 7, the RMSE and MAE values for both Ta and Tmrt in summer and winter were below their respective maximum thresholds [65]. Therefore, ENVI-met is considered reliable for subsequent analysis of the effects of landscape elements on PET.

## 3. Results

### 3.1. Characteristics of Outdoor Activities and Thermal Benchmarks

#### 3.1.1. Outdoor Activity Patterns

**Figure 3:**
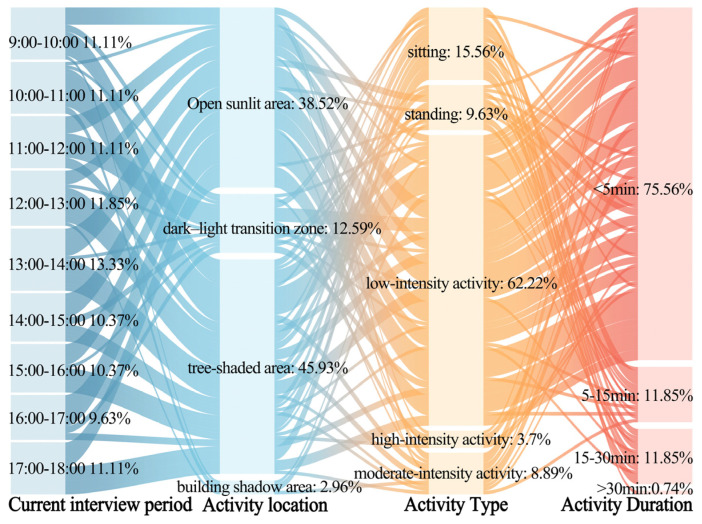
The activity status of the respondents in the outdoor leisure plaza of the universities during the summer.

**Figure 4:**
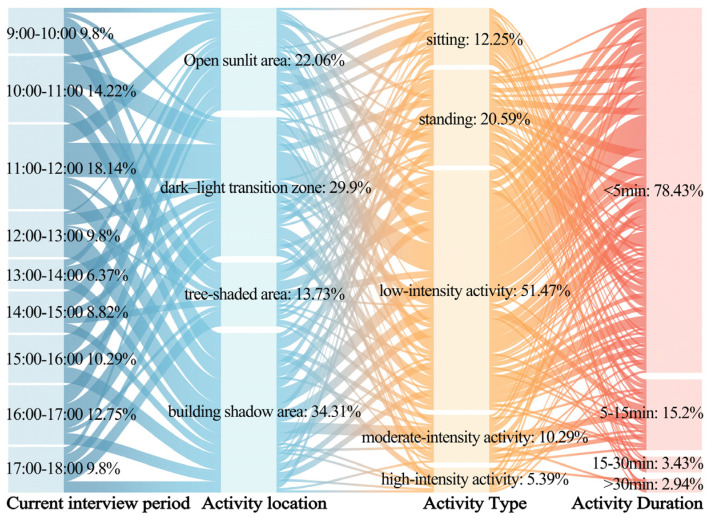
The activity status of the respondents in the outdoor leisure plaza of the universities during the winter.

Figure 3 and Figure 4 illustrate the primary activity characteristics of the respondents in summer and winter, respectively. Notable seasonal differences were observed in the activity time periods and locations within the plaza, while activity types and durations exhibited similar characteristics across both seasons.

In summer, the main activity periods were from 13:00 to 14:00 and from 17:00 to 18:00, during which approximately 50% of the respondents were active in tree-shaded areas and building-shaded areas. This indicates that nearly half of the respondents preferred to stay in shaded areas to reduce heat exposure under high temperatures. In winter, the main activity periods were from 11:00 to 12:00 and from 16:00 to 17:00, with over 50% of the respondents active in open sunlit areas and sun–shade transition zones. This suggests that more than half of the respondents tended to stay in sunlit areas to alleviate cold stress in low temperatures.

Additionally, in both summer and winter, over 85% of the respondents engaged in standing, sitting, or low-intensity physical activities, and more than 75% had activity durations of less than 5 min. This indicates that the respondents had short stays and limited activity types in the plaza.

These findings not only provide data as support for developing a time- and season-weighted thermal comfort evaluation mechanism but also offer empirical evidence for proposing nature-based solutions for the site.

#### 3.1.2. Neutral PET Range

**Figure 5:**
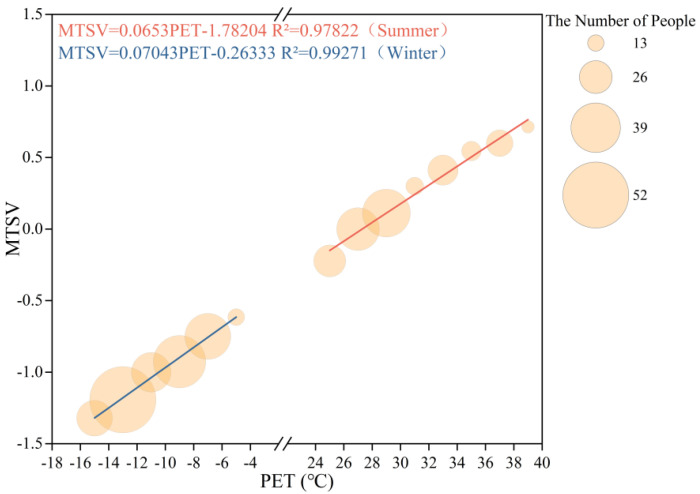
The linear relationship between the MTSV and PET for the respondents in the outdoor leisure plaza of the universities during the summer and winter seasons.

The neutral PET range was determined using the temperature frequency method (bin method), in which PET values were grouped in 2 °C intervals. The groups with fewer than three respondents were excluded. A linear regression was then performed with the central PET value of each group as the independent variable and the corresponding mean thermal sensation vote (MTSV) as the dependent variable, as shown in Figure 5.

The results show that when –0.5 ≤ MTSV ≤ 0.5, the neutral PET range for the respondents in the plaza was 19.63 ≤ PET ≤ 34.94 °C in summer. Under the same MTSV condition, the neutral PET range in winter was –3.36 ≤ PET ≤ 10.84 °C.

#### 3.1.3. 90% Acceptable PET Range

**Figure 6:**
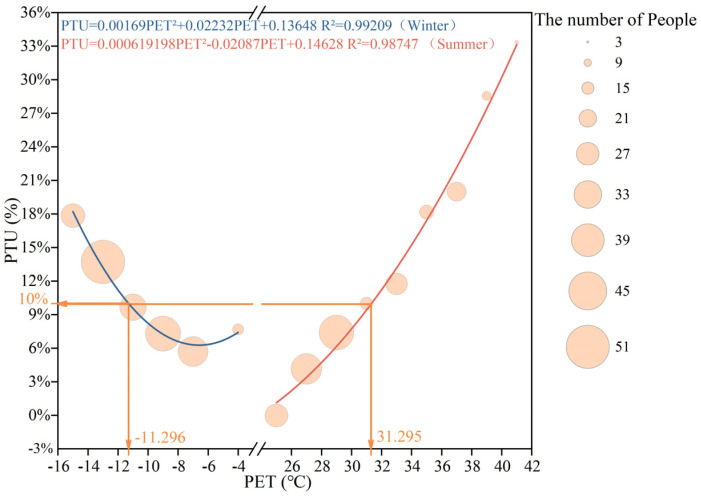
The 90% acceptable PET range for the respondents in the outdoor leisure plaza of the universities during the summer and winter seasons.

Similarly, the 90% acceptable PET range was determined using the temperature frequency method (bin method). A quadratic regression was performed with the central PET value of each group as the independent variable and the corresponding MTSV as the dependent variable, as shown in Figure 6.

The results show that when the Percentage of Thermal Unacceptability (PTU) equals 10%, the upper limit of the 90% acceptable PET range in summer is PET = 31.3 °C, and the lower limit in winter is PET = −11.3 °C. Compared to the neutral PET range, the 90% acceptable PET range is narrower, which is consistent with the findings of Cheung and Jim [71].

**Figure 7:**
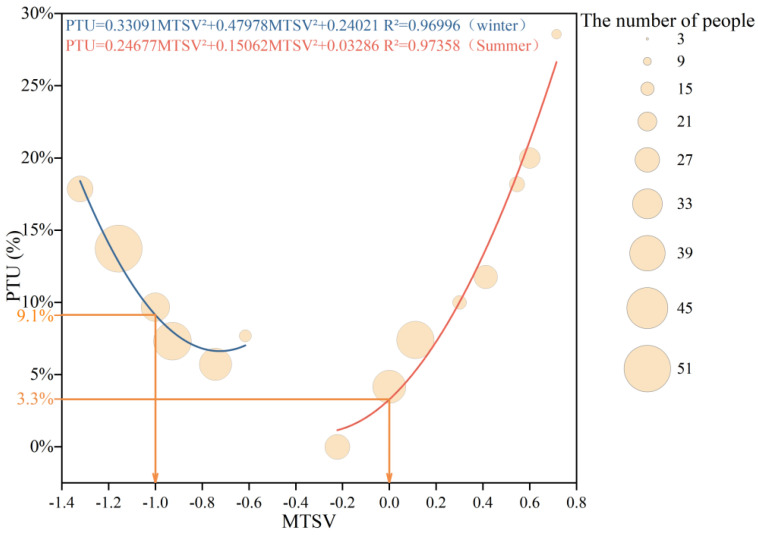
Quadratic regression model between MTSV and PTU in winter and summer seasons of a university outdoor leisure plaza.

To calibrate the PET thresholds for the respondents in the plaza, this study conducted a quadratic regression between the MTSV and PTU for winter. When the MTSV = −0.5, the PTU was 8.3%, which is close to the 10% threshold, further supporting the reliability of using PTU = 10% as the boundary for the neutral PET range. When the MTSV values were −1.5 and −2.5, the corresponding PTU values were 26.5% and 110.8%, respectively, as shown in Figure 7. Given that the PTU cannot exceed 100%, PTU values of 26.5% and 100% were substituted into the winter PET-PTU regression equation (Figure 6), yielding corresponding PET values of −17.54 °C and −30.15 °C.

Similarly, in summer, when the MTSV values were 0.5, 1.5, and 2.5, the corresponding PTU values were 17.0%, 81.4%, and 195.2%, respectively, as shown in Figure 7. Based on the assumption that PTU = 10% defines the neutral PET range and that the maximum PTU is capped at 100%, PTU values of 81.4% and 100% were substituted into the summer PET-PTU regression equation, resulting in PET values of 53.74 °C and 57.6 °C, respectively.

Finally, as shown in Table 8, calibrating the outdoor thermal sensation categories of the respondents in both summer and winter established a theoretical basis for evaluating the seasonal outdoor thermal environment in the plaza.

### 3.2. Effects of Landscape Elements on Outdoor Thermal Comfort

#### 3.2.1. Ranking of Main Effects

To assess the influence of greening layout (A), plant type (B), and surface albedo (C) on the PET, the mean PET values (i.e., $\bar{{PET}_{AL}},\;\bar{{PET}_{BL}},\;\bar{{PET}_{CL}}$) were calculated for each factor at four levels (i.e.,L = 1, 2, 3, 4) during summer and winter. The range of the PET values (i.e., $R_{AL}$, $R_{BL}$, $R_{CL}$) for each factor was then used to determine its relative impact across different time periods. The calculation procedure is detailed in Appendix A.4.

The results show that the plant type (B) had the greatest impact on the he PET during both summer periods (13:00–14:00 and 17:00–18:00) and during winter from 11:00 to 12:00. In contrast, the greening layout (A) had the most significant influence on the PET during winter from 16:00 to 17:00.

**Figure 8:**
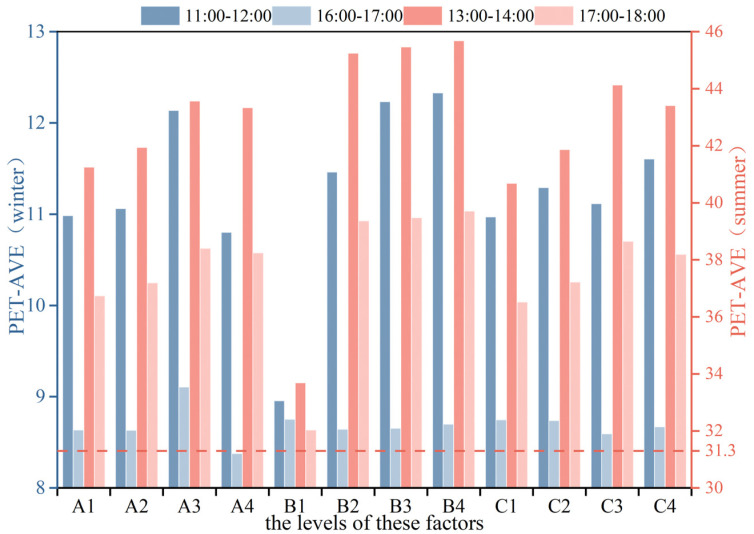
Mean PET values for each time period in summer and winter under different levels of landscape elements.

As illustrated in Figure 8, during winter, the mean PET values for all levels of each landscape element remained within the neutral PET range (i.e., PET ≥ −11.3 °C), while in summer, they all fell within the slightly warm range (i.e., 31.3 °C < PET ≤ 53.7 °C).

**Figure 9:**
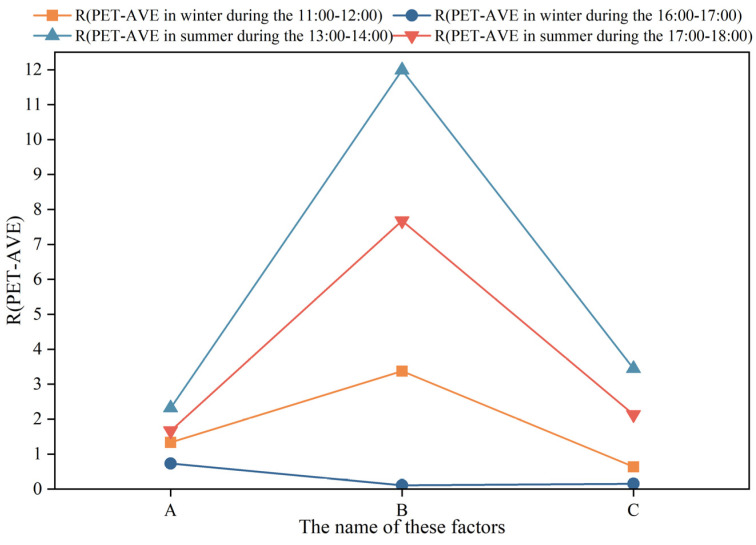
PET range for each time period in summer and winter under different levels of each landscape element.

In addition, all landscape elements exhibited a greater influence on the PET in summer compared to winter. As shown in Figure 9, the greatest impact on the PET occurred during the summer period from 13:00 to 14:00, followed by the summer period from 17:00 to 18:00 and the winter period from 11:00 to 12:00. The least influence was observed during the winter period from 16:00 to 17:00.

#### 3.2.2. Significance and Contribution Ranking

One-way analysis of variance (ANOVA) was used to calculate the sum of squares (SSA, SSB, SSC) for the greening layout (A), plant type (B), and surface albedo (C), each at four levels (L = 1, 2, 3, 4), during different activity periods in summer and winter. F-tests were then conducted to assess the statistical significance of each factor’s effect on the PET. For example, the calculation steps for the significance of the plant type (B) on the PET during the summer period from 13:00 to 14:00 are detailed in Appendix A.5. The results indicate that the greening layout (A) significantly influenced the PET in winter, while the plant type (B) had a significant effect on the PET in summer.

**Figure 10:**
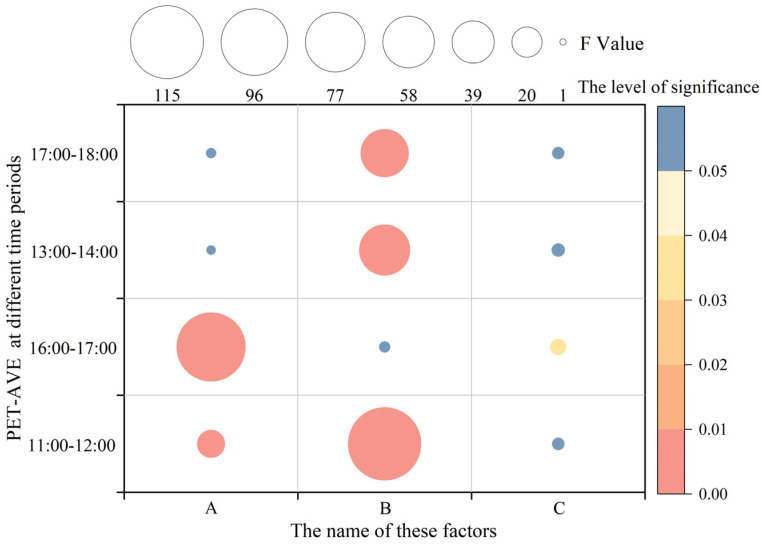
Analysis of F-values and significance of each landscape element on PET across time periods in summer and winter.

As shown in Figure 10, the plant type (B) had a statistically significant effect on the PET during both summer periods (13:00–14:00 and 17:00–18:00) and the winter period from 11:00 to 12:00.

Specifically, during winter from 11:00 to 12:00, both the greening layout (A) and plant type (B) significantly influenced the PET. During winter from 16:00 to 17:00, the greening layout (A) and surface albedo (C) showed significant effects. In summer, during both 13:00–14:00 and 17:00–18:00, the plant type (B) was the only factor with a significant impact on the PET.

The calculation steps for the contribution rates of each landscape element to the PET during different activity periods in summer and winter are presented in Appendix A.6 [60]. As there are two primary activity periods in both seasons, the contribution weights of the greening layout (A), plant type (B), and surface albedo (C) to the PET in each season were set to 0.5. Given that summer in Hohhot lasts for 40 days and winter for 182 days, the combined seasonal weight for the contribution rate calculations was normalized to 1. Accordingly, the weights for summer and winter were 0.82 and 0.18, respectively, for the greening layout (A), plant type (B), and surface albedo (C).

**Figure 11:**
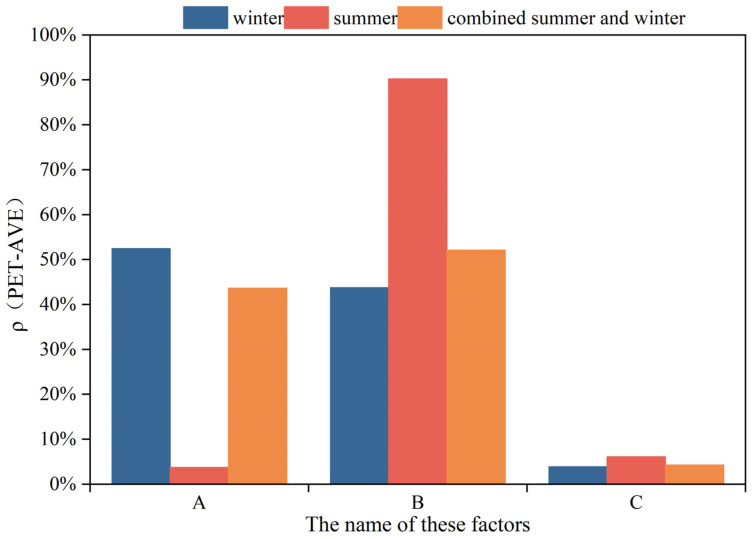
Contribution rates of each landscape element to PET in summer and winter.

As shown in Figure 11, the overall contribution ranking of the landscape elements to the PET is as follows: plant type (B) > greening layout (A) > surface albedo (C). Specifically, in summer, the ranking was plant type (B) > surface albedo (C) > greening layout (A), whereas in winter, the order shifted to greening layout (A) > plant type (B) > surface albedo (C).

### 3.3. Optimal Experimental Scheme Selection

**Figure 12:**
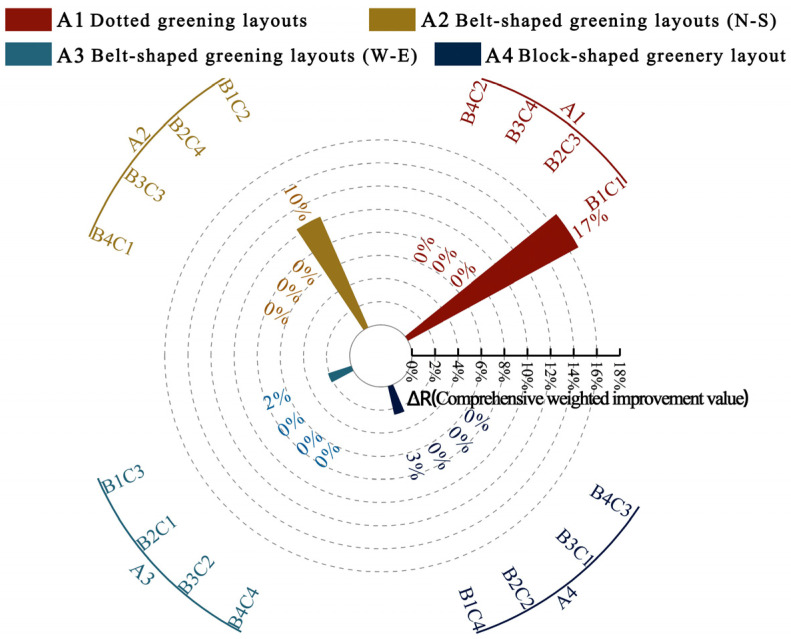
Weighted improvement in the proportion of activity areas within the neutral PET range in summer and winter for each experimental scheme, relative to the original site.

As shown in Figure 12, the experimental scheme A1B1C1 achieved the greatest weighted increase in the proportion of activity areas within the neutral PET range across both summer and winter, followed by A2B1C2 and A4B1C4, with A3B1C3 showing the least improvement. The calculation steps for determining the optimal scheme are detailed in Appendix A.7.

Since both the original scheme and the experimental schemes were within the neutral PET range during winter, there was no increase in the proportion of activity areas within the neutral PET range in winter compared to the original scheme. Therefore, this study comprehensively considered the weighted improvement in the proportion of activity areas within the neutral PET range across both summer and winter seasons, relative to the original scheme, and ultimately determined the optimal scheme.

### 3.4. Nature-Based Solution

Based on the integrated effects of the vegetation layout, plant type, and surface albedo on outdoor thermal comfort in both summer and winter, the following nature-based solution is proposed:

(1) Greening layout: The site perimeter adopts a strip-type greening layout, while block-type green spaces are arranged in the interior. Specifically, two mutually perpendicular strip green belts are set along the southwest, northwest, and northeast sides, with the western side supplemented by strip green belts and green walls.

This perpendicular arrangement of greenery helps mitigate the penetration effects of Hohhot’s prevailing southwest, northwest, and southeast winds in winter, as well as the prevailing northeast and west winds in summer [42], thereby further enhancing wind protection [56]. The strip-shaped green space and the green wall on the west side of the site not only preserve the integrity of the summer east–west ventilation corridor but also effectively reduce the intrusion of strong summer winds and the impact of cold winter winds.

(2) Plant configuration: The planting strategy follows the principle of alternating evergreen and deciduous trees along the site perimeter, while the interior block-type green spaces adopt a structure of trees surrounding shrubs. Along the peripheral strip belts, *Populus nigra* (deciduous) and *Pinus tabuliformis* (evergreen) are alternately planted to reduce wind speed and improve outdoor thermal comfort. In the interior block-type green areas, tall *Populus nigra* are planted around the perimeter to provide shade and enhance cooling through transpiration. Overlapping canopy areas are interplanted with the low deciduous tree Prunus triloba, and the inner sections are filled with the shrub border privet, forming a multilayered vegetative belt where trees surround shrubs.

Yang et al.’s research indicated that the planting of trees surrounding shrubs in block-shaped green spaces can significantly improve outdoor thermal comfort in summer [72]. This study also found that black poplar contributes the most to the thermal comfort of the site in both summer and winter among different plant types. Tall trees not only effectively reduce solar radiation in the site during summer but also enhance cooling and humidification through intense transpiration [73]. In winter, the shedding of leaves allows more solar radiation to penetrate the site [74]. Therefore, this study extensively planted black poplars on the outer side of block-shaped green spaces to improve the thermal environment at the site.

(3) The surface materials were selected using the approach of “low reflectance materials for dynamic areas and medium reflectance materials for static areas.” The outer running track and core activity area of the site are paved with red plastic and dark granite, both with a reflectivity of 0.2, which can effectively reduce the average radiant temperature within the site [35]. In contrast, the rest and reading areas are paved with wooden materials with a reflectivity of 0.4 to reduce glare and create a warm, tranquil environment conducive to reading and social interaction.

**Figure 13:**
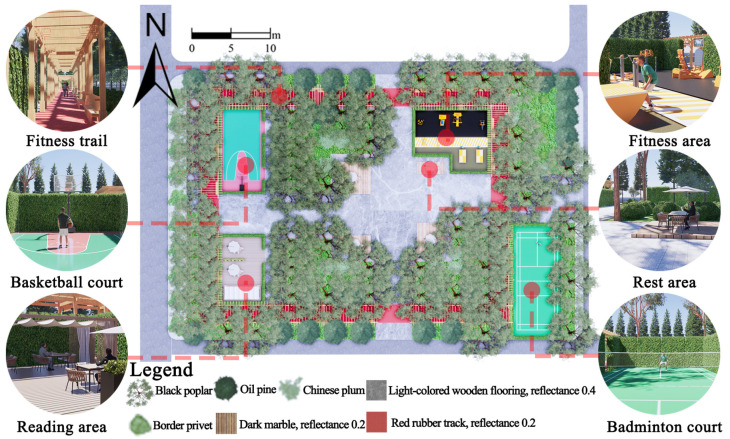
Plan layout and local renderings after site renovation.

As shown in Figure 13, the optimized site features a circular fitness trail along its perimeter, suitable for moderate-intensity activities. A basketball court is located in the northwest corner and a badminton court in the southeast corner, both designated for high-intensity activities. A fitness zone is situated in the northeast corner to accommodate diverse exercise needs. In contrast, a reading area and a resting zone are located in the southwest corner and central area, respectively, catering to low-intensity activities.

This spatial arrangement ensures a rational distribution of activity zones, ranging from high to low intensity.

**Figure A1:**
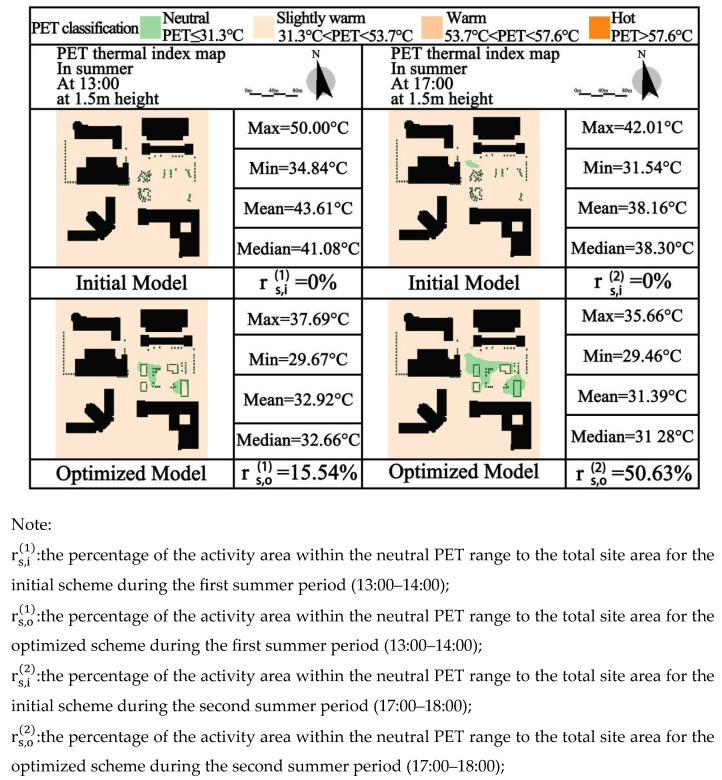
PET distribution map in summer before and after site renovation.

**Figure A2:**
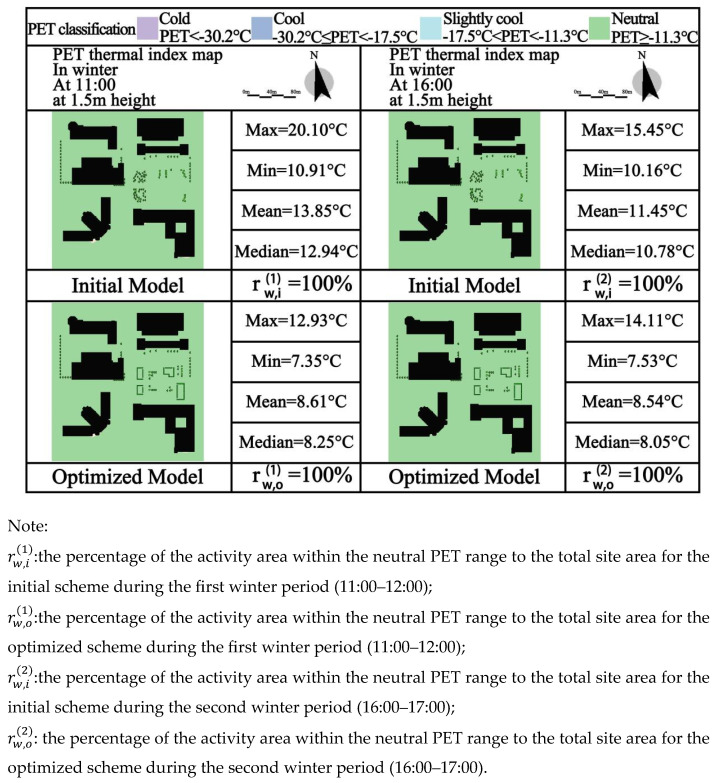
PET distribution map in winter before and after site renovation.

Following optimization, the area within the neutral PET range in summer expanded significantly, primarily in zones with strip-type green layouts, continuous *Populus nigra* planting, and low-albedo surfaces, particularly in the northwest and southeast corners (see Figure A1 and Figure A2 in Appendix A.8 for details). This demonstrates that adjustments to the greening layout, plant type, and surface material effectively improved the thermal comfort conditions.

By contrast, both the original and optimized layouts remained within the neutral PET range in winter, benefiting from the original design’s provision of ample solar access and reduced wind speed. Therefore, in cold regions, winter optimization should focus more on maximizing solar exposure and mitigating wind chill, rather than emphasizing shading and evaporative cooling.

Further calculations showed that the optimized scheme increased the proportion of activity areas within the neutral PET range by 6.0% across both seasons, with a 33.1% increase in summer. No improvement was observed in winter, as the original design already satisfied the neutral PET conditions. These results confirm the effectiveness of the proposed scheme in enhancing seasonal thermal comfort in severely cold regions, achieving the coordinated optimization of the outdoor thermal environment for both summer and winter.

## 4. Discussion

### 4.1. Comparison of Thermal Benchmarks in Representative Cold-Climate Cities

**Figure 14:**
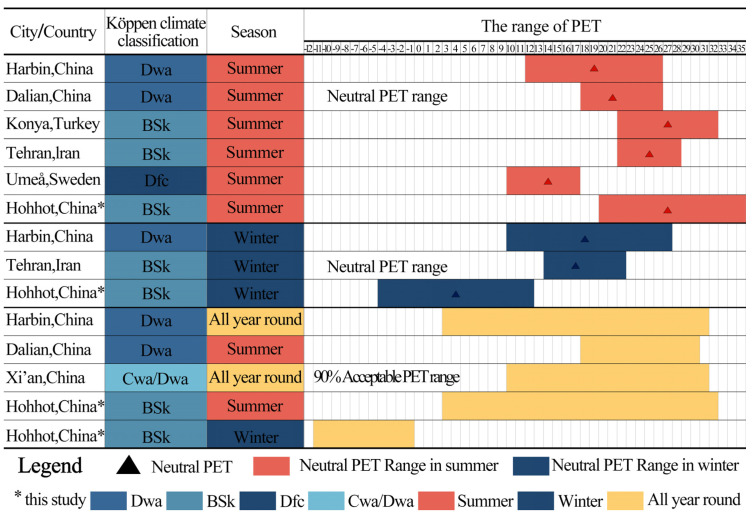
Comparison of thermal benchmarks in representative cold-climate cities.

The study compares the outdoor thermal comfort results of Hohhot in both summer and winter with typical cities in cold climate regions (Harbin, China [75]; Dalian, China [76]; Konya, Turkey [77]; Tehran, Iran [78]; Umeå, Sweden [79]; Xi’an, China [80]), analyzing the differences and causes of a neutral PET and its range compared to the 90% acceptability PET range. As shown in Figure 14, Hohhot has the highest neutral PET in summer, while Umeå, Sweden, has the lowest.

However, Hohhot has the lowest neutral PET in winter, while Harbin has the highest. The extreme values of neutral PET in Hohhot in both summer and winter are likely related to the survey dates and exposure duration. In fact, Chen et al. found that, under the same PET, the average thermal sensation vote at the end of winter is about one scale higher than that at the beginning of winter [75]. These findings suggest that the longer the respondents are exposed to the same season, the lower their psychological expectation, which further reduces their response to thermal stimuli [81]. Therefore, although Hohhot’s annual temperature range is smaller than Harbin’s, the respondents in Hohhot exhibit higher neutral PET in summer and lower neutral PET in winter, indicating that the respondents in Harbin have relatively weaker outdoor thermal adaptation under shorter exposure durations.

Hohhot has the widest neutral PET range in summer, while Tehran has the narrowest. However, Harbin has the widest neutral PET range in winter, with Tehran still having the narrowest. This is related to the fact that the average temperature in the hottest month in Hohhot is lower than in Harbin, and the temperature variation is greater in Hohhot than in Harbin, which leads to a wider neutral PET range for the respondents in Hohhot compared to those in Harbin. Moreover, the respondents in Harbin are more likely to accept a lower neutral PET than those in Hohhot. A similar conclusion was reached by Mi et al. [80]. Additionally, Harbin has a lower average temperature in the coldest month and a longer winter duration, which reduces the respondents’ sensitivity to low temperatures, making its neutral PET range in winter the widest in the above study. Yin et al. also observed a similar phenomenon [82].

Hohhot had the widest 90% acceptable PET range in summer, whereas Dalian had the narrowest. Furthermore, Dalian had the lowest lower limit of the 90% acceptable PET range in winter. These variations may be attributed to differences in the actual climatic conditions during the survey periods across cities in cold regions, even within the same season, which could influence the width of the respondents’ acceptable PET ranges [83]. Moreover, the 90% acceptable PET range in summer for the respondents in Dalian was broader than their neutral PET range. This may be related to the respondents’ prior thermal experiences, which could have enhanced their psychological tolerance to heat [84].

### 4.2. Analysis of the Specific Effects of Landscape Elements on the PET

This study further analyzed the specific impacts of the greening layout, plant type, and surface albedo—as well as their respective levels—on the PET in order to explore their regulatory mechanisms on the outdoor thermal environment during different periods in summer and winter.

(1) Plant Type

As indicated in Section 3.2.2, the plant type had the highest contribution rate to the PET in both summer and winter, with *Populus nigra* (B1) showing the most significant effect. In summer, B1 yielded the lowest average PET. Given that plant characteristics, such as height, canopy shape, leaf area index, and permeability, can influence the outdoor thermal environment [85], *Populus nigra*, as the tallest and widest-canopy tree species in the plaza, provided dense foliage that effectively blocked substantial shortwave radiation during midday, while enhancing transpiration. These factors led to a stronger cooling effect at midday compared to later in the afternoon [86].

In winter, B1 also produced the lowest average PET between 11:00 and 12:00, but the highest between 16:00 and 17:00. Perini et al. noted that although *Populus nigra* is a deciduous tree, its tall trunk and broad canopy can still obstruct part of the incoming shortwave radiation during late morning hours, resulting in the lowest PET during that time [87]. In contrast, between 16:00 and 17:00, as solar radiation declined, *Populus nigra* absorbed longwave radiation, thereby increasing the mean radiant temperature and leading to the highest PET in that period [88].

(2) Greening Layout

The greening layout (A) ranked second after the plant type (B) in terms of contribution to the PET in both seasons. In summer, the dotted-shaped layout (A1) resulted in the lowest average PET, while the strip-type layout, aligned in the N–S direction (A2), produced the highest PET in winter. In summer, the cooling capacity of each dotted-shaped green patch was sufficient. As the landscape fragmentation increased, additional shading was provided by adjacent tree-lined roads, further enhancing the cooling effect [56].

In winter, although the N–S strip green layout aligns with Hohhot’s prevailing winter wind direction, its positioning in the central part of the site and the shielding effect of buildings on the windward side limited the shading impact of vegetation. This allowed most activity areas to receive sufficient radiative heat from the surrounding environment, thereby improving thermal comfort.

(3) Surface Albedo

Surface albedo (C) had the lowest contribution to the PET in both seasons. In summer, materials with a reflectance of 0.2 (C1) resulted in the lowest average PET. Chu et al. found that although high-albedo materials can reduce solar absorption by the pavement, they also increase the mean radiant temperature through multiple reflections, thereby reducing thermal comfort [37]. In addition, plant shading and transpiration reduce the relative contribution of surface albedo to the PET is smaller than that of the plant type and greening layout [36].

In winter, materials with an albedo of 0.5 (C4) showed the highest PET between 11:00 and 12:00, while materials with an albedo of 0.2 (C1) showed the highest PET between 16:00 and 17:00. The former can be attributed to the increased reflection of solar radiation by high-albedo materials, which elevated the average radiant temperature and, consequently, the PET—an observation consistent with Taleghani’s findings [89]. The latter can be explained by the ability of low-albedo materials to absorb solar radiation throughout the day and re-radiate it as longwave radiation in the evening, thereby increasing the PET during the late hours [90].

### 4.3. Study Limitations

Although this study has made preliminary progress in the thermal comfort evaluation and optimization of outdoor plazas at universities in cold regions based on landscape elements, there are still limitations in aspects such as numerical simulations, greenery configuration, plant characteristics, and the interactions of landscape elements, which need to be addressed in future research.

(1) Limitations in the numerical simulation due to the temporal scope and climatic conditions.

This study employed simulations based on typical summer and winter days in Hohhot. However, it did not cover full diurnal cycles or multi-day weather conditions, nor did it account for nighttime thermal comfort among outdoor users [61].

(2) The scope of the greening layout and planting strategies.

The analysis was based on regular rectangular green space layouts and did not evaluate the thermal regulatory potential of irregularly shaped green spaces [37]. Additionally, the relationship between the green space orientation and prevailing wind directions [56], as well as the influence of planting parameters—such as plant spacing [72], planting position [91], and the placement order of trees and shrubs [92]—on cooling performance, were not addressed.

(3) The impact of plant characteristics on outdoor thermal comfort requires further validation.

This study selected plant species based on the plant heights and their proportion in the site, but it has not considered a wider variety of plants in the outdoor leisure plaza of the university in Hohhot. Additionally, it has not compared the effects of different plant species of the same type, taking into account factors such as canopy geometry [93], vertical dimensions (e.g., tree height [94], trunk height [95], and crown height [96]), LAI [97], and biological characteristics (e.g., transpiration [98] and evaporation [99]). These factors have not yet been explored for their specific impact on outdoor thermal comfort.

(4) The lack of exploration of the interaction effects among landscape elements.

This study focused solely on the main effects of the greening layout, plant type, and surface albedo on the PET, without investigating the potential synergistic mechanisms among these factors [100,101,102], such as the combined influence of vegetation configuration and surface materials on thermal comfort.

## 5. Conclusions

This study focuses on an outdoor recreational plaza at a university in Hohhot, aiming to establish its thermal benchmarks for both summer and winter, evaluate the seasonal effects and significance of vegetation layout, plant type, and surface albedo on thermal comfort, identify the optimal combination that balances thermal comfort across both seasons, and propose a nature-based solution suitable for severely cold regions. The main conclusions are as follows:

(1) The thermal benchmarks for the outdoor leisure plaza at the university in Hohhot were clarified for both summer and winter. In winter, the lower limit of the neutral PET range is −11.3 °C, and in summer, the upper limit of the neutral PET range is 31.3 °C.
(2) The study revealed the overall contribution of landscape elements to seasonal outdoor thermal comfort and identified the optimal combination. The overall contribution of the landscape elements to the PET in both summer and winter ranks as follows: plant type > greenery layout > surface albedo. The combination of “dot-shaped greenery layout + black poplar + surface albedo of 0.2” was selected as the optimal configuration for ensuring thermal comfort in both summer and winter.
(3) A nature-based solution was proposed that integrates thermal comfort and functional needs. Compared to the initial configuration, the optimized scheme achieved a 6.0% increase in the proportion of activity area within the neutral PET range across both summer and winter seasons.

## Figures and Tables

**Table 1:** Measurement range and accuracy of these instruments.

Device Names	Measurement Parameters
Cup anemometer	Wind speed: Measurement range: 0–70 m/s, resolution: 0.1 m/s, accuracy: ±(0.3 + 0.03 V) m/s
wind vane	Wind direction: Range: 0–360°, resolution: 1°, accuracy: ±3°
Multi-parameter Stevenson screen	Ambient temperature: Range: −40 to +80 °C, resolution: 0.1 °C, accuracy: ±0.2 °C Ambient humidity: Range: 0–100% RH, resolution: 0.1% RH, accuracy: ±2% (≤80% RH), ±5% (>80% RH)
Pyranometer	Solar radiation: Range: 0–2000 W/m^2^, resolution: 1 W/m^2^, accuracy: ≤5%
JTR04 globe thermometer	Globe thermometer: Temperature range: 10–85 °C, temperature accuracy: ±0.5 °C, resolution: 0.1 °C, globe diameter: 150 mm, emissivity: >0.95

**Table 2:** Orthogonal experimental factors and their levels.

	Factor	Greening Layouts (A)	Plant Types (B)	Surface Albedo (C)
Level				
1		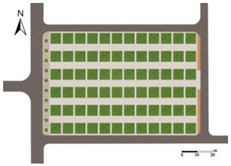	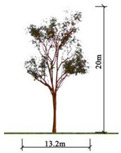	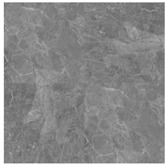
		Dotted-shaped greening layouts	Black poplar (*Populus nigra*)	0.2
2		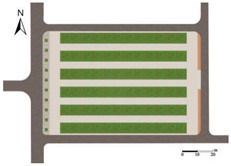	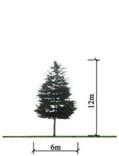	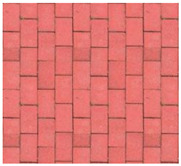
		Belt-shaped greening layouts (N-S)	Oil pine (*Pinus tabuliformis*)	0.3
3		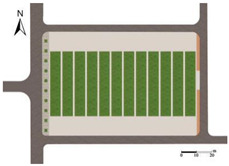	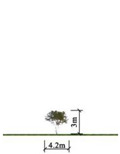	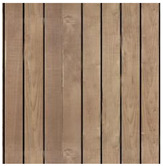
		Belt-shaped greening layouts (W-E)	Chinese plum (*Amygdalus triloba*)	0.4
4		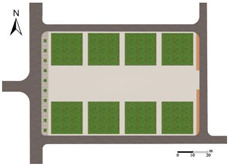	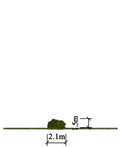	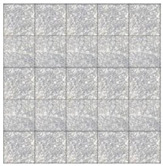
		Block-shaped greenery layout	Border privet	0.5

**Table 3:** Characteristic parameters of the selected plant species in the plaza.

Plant Name	Plant Type	Plant Height (m)	Canopy Diameter (m)	LAI [57]	Proportion of Total Plant Count (%)
Black poplar	Deciduous tree	20	13.2	3.37	10.4%
Chinese Pine	Evergreen tree	12	6.0	5.38	40.5%
Chinese Plum	Deciduous tree	3	4.2	2.34	29.4%
Privet	Deciduous shrub	1.5	2.1	2.54	11.7%

**Table 4:** Partial correlation significance and correlation coefficients between PET and different outdoor thermal environment parameters during summer and winter.

	Partial Correlation	PET and Ta		PET and RH		PET and Va		PET and Tmrt	
Season		P	R	P	R	P	R	P	R
Summer		0.000	0.628	0.218	0.108	0.000	−0.963	0.000	0.997
Winter		0.000	0.358	0.136	0.106	0.000	−0.784	0.000	0.413

Note: P represents the significance (two-tailed), and R represents the correlation coefficient.

**Table 5:** ENVI-met boundary condition settings.

Parameter	Summer (29 June 2024)	Winter (7 December 2024)
Start time	7:00	7:00
Simulation duration	11 h	11 h
Daily maximum/minimum temperature (°C)	27.37 °C/18.24 °C	−2.44 °C/−11.31 °C
Daily maximum/minimum relative humidity (%)	56.65%/34.94%	61.73%/37.50%
Specific humidity at 2500 m (g/kg)	8.57	0.78
Constant wind speed at 10 m (m/s)	2.04	1.17
Constant wind direction at 10 m (°)	180	180
Ground microscale roughness length (m)	1.00	1.00
Low cloud cover (0–8)	0	0
Middle cloud cover (0–8)	6	2
High cloud cover (0–8)	1	0

**Table 6:** Physiological characteristics of respondents in winter and summer.

Parameter	Summer	Winter
Age (y)	22	22
Gender	M	M
Weight (kg)	67.49	67.66
Height (m)	1.73	1.75
Body posture	Standing	Standing
Walking speed (m/s)	1.2	1.2
Static outdoor clothing insulation (clo)	0.4	1.72
Indoor clothing insulation (clo)	0.4	1.2
Metabolic rate during activity (M)	142.37	142.41

**Table 7:** Error analysis between simulation results and measured microclimate data.

	Statistical Indicators	RMSE	MAE
Meteorological Parameters			
Ta-summer (°C)		0.6	0.5
Ta-winter (°C)		1.1	0.7
Tmrt-summer (°C)		13.8	12.7
Tmrt-winter (°C)		13.7	10.4

Note: (RMSE-Ta)max = 4.3 °C; (MAE-Ta) max = 3.67 °C; (RMSE-Tmrt) max = 13.9 °C; (MAE-Tmrt) max = 12.7 °C.

**Table 8:** Calibrated PET classification for university students in Hohhot.

Stress Category	PET Classification in Summer	PET Classification in Winter
Hot	PET > 57.6 °C	—
Warm	53.7 °C < PET ≤ 57.6 °C	—
Slightly warm	31.3 °C < PET ≤ 53.7 °C	—
Neutral	PET ≤ 31.3 °C	PET ≥ −11.3 °C
Slightly cool	—	−17.5 °C ≤ PET < −11.3 °C
Cool	—	−30.2 °C ≤ PET < −17.5 °C
Cold	—	PET < −30.2 °C

## Data Availability

The original contributions presented in this study are included in the Appendix A of this article. In addition, the download link for the RayMan Pro software used to calculate the PET is as followsfile shared via cloud storage: RayManPro.zip; link: https://pan.baidu.com/s/1K4XqN89dI_gFIB5I-fh-nQ?pwd=1234; extraction code: 1234. Shared by Baidu Netdisk Super Member v9. If you have any questions, please contact the corresponding author.

## Abbreviations

The following abbreviations are used in this manuscript:

PMV	Predicted Mean Vote
MAPE	Mean Absolute Percentage Error
TMD	Typical meteorological day
Ta	Air temperature
RH	Relative humidity
Va	Wind speed
Tg	Globe temperature
PET	Physiological Equivalent Temperature
MEMI	Munich Energy-balance Model for Individuals
MTSV	Mean thermal sensation vote
PTU	Percentage of Thermal Unacceptability

## Appendix A

### Appendix A.1

Summer Thermal Comfort Survey for Outdoor Leisure Squares on University Campuses

Note: This section is to be completed by the investigator and must be filled out at the time of the interview.

Survey Date	___ June 2024
Time of completion	: - :

Time period of the interview [Single choice] *:

□9:00–10:00	□10:00–11:00	□11:00–12:00	□12:00–13:00
□13:00–14:00	□14:00–15:00	□15:00–16:00	□16:00–17:00 □17:00–18:00

Mark your current location

(Please use an “X” on the image to indicate your specific location within the plaza):

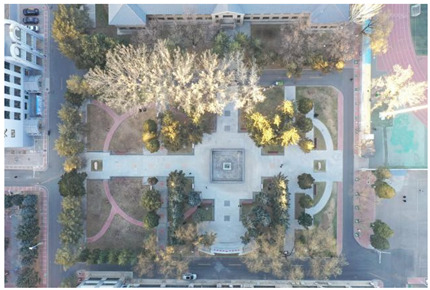

Area you are currently in during the interview [Single choice] *:

□Open sunny area □Light–shade interface □Building shaded area □Tree-shaded area

Part I: Personal Basic Information Survey (Respondents may answer this section repeatedly, but each response must be at least 30 min apart)

1. Gender: □ Male□ Female;

2. Age: _______________(years);

3. Height:__________(m);

4. Weight:_______________(kg);

5. Clothing—Upper body [Multiple choices depending on situation] *:

□Sleeveless top (0.12clo)	□Short-sleeved T-shirt (0.19clo)	□Long-sleeved T-shirt (0.25clo)	□Short-sleeved shirt/dress (0.29clo)	□Long-sleeved sports shirt (0.34clo)	□Long-sleeved outerwear (0.36clo)

6. Clothing—Lower body [Multiple choices depending on situation] *:

□Underwear (0.03clo)	□Sports shorts (0.08clo)	□Thin trousers (0.15clo)	□Ultra-short skirt (incl. shorts 0.06clo)	□Shorts (0.14clo)	□Long skirt (0.23clo)

7. Footwear [Multiple choices depending on situation] *:

□Ultra-short socks(0.02clo)	□Ankle socks (0.03clo)	□Shoes (summer, autumn 0.02clo)	□Leather shoes (0.04clo)	□Sandals (incl. flip-flops 0.02clo) (0.02clo)

8. Your primary outdoor activity [Single choice] *:

□ Sitting (e.g., sunbathing, using phone, reading, chatting) (60W) (M < 1.2 met) □ Standing (e.g., reading info boards, light talking) (70W) (M < 1.2 met) □ Low-intensity exercise (e.g., walking < 1.2 m/s, Tai Chi, slow dancing) (150W) (1.2 ≤ M < 2.6 met)
□ Moderate-intensity exercise (e.g., brisk walking, fitness, dancing) (220W) (2.6 ≤ M < 3.8 met) □ High-intensity exercise (e.g., running, ball games) (360W) (M > 3.8 met)

Part II—Outdoor Space Use and Perception Survey

1. How long have you lived here? [Single choice] *:

□<6 months	□6 months–1 year	□1–3 years	□More than 3 years

2. On weekends/holidays, when do you most often stay in this space? [Multiple choices]*:(Note: Select at least 1 and up to 10 options)

□8:00–9:00	□9:00–10:00	□10:00–11:00	□11:00–12:00	□12:00–13:00
□13:00–14:00	□14:00–15:00	□15:00–16:00	□16:00–17:00	□17:00–18:00

3. What activity were you mainly doing 15 min ago? [Single choice] *:

□Lying down □Sitting □Low-intensity exercise (walking, Tai Chi, yoga, etc.) □Moderate-intensity exercise (brisk walking, jogging, etc.) □High-intensity exercise (ball games, competitive sports, etc.)

4. What kind of environment were you in 15 min ago? [Single choice] *:

□Indoors with air conditioning □Naturally ventilated indoors □Shaded outdoor space □Sunny outdoor space

5. What is your purpose here? [Single choice] *:

□Rest □Exercising □Socializing □Enjoy scenery □Reading □Group activity □Passing by

6. Frequency of outdoor activities in this space [Single choice] *: (Note: More than once counts as multiple times)

□Daily □Several times a week □Weekly □Occasionally □Rarely

7. Duration of each outdoor visit [Single choice] *:

□<5min □5–15min □15–30min □>30min

8. Where do you mainly stay in the space? [Multiple choices] *

□Open sunny area □Tree-shaded area/building shade □Seating/rest area □Windless area □Constantly moving (if selected, no need to choose others; otherwise, select at least 1 and up to 3 options)

Part III—Outdoor Perception and Preferences Survey

1. How do you feel about the current thermal environment? [Single choice] *:

□Very cold (−3)	□Cold (−2)	□Cool (−1)	□Neutral (0)	□Slightly warm (+1)	□Warm (+2)	□Very hot (+3)

2. How comfortable do you feel now? [Single choice] *:

□Comfortable (0)	□Slightly uncomfortable (+1)	□Uncomfortable (+2)	□Very uncomfortable (+3)

3. To what extent do you accept the current thermal environment? [Single choice]*

□Fully acceptable (0)	□Slightly acceptable(−1)	□Slightly unacceptable(−1)	□Very unacceptable(−1)

4. Regarding current air temperature, you prefer: [Single choice] *

□Lower (−1)	□No change (0)	□Higher (+1)

5. Regarding current solar radiation, you prefer: [Single choice] *

□Lower (−1)	□No change (0)	□Higher (+1)

6. Regarding current wind speed, you prefer: [Single choice] *

□Lower (−1)	□No change (0)	□Higher (+1)

7. Regarding current relative humidity, you prefer: [Single choice] *

□Lower (−1)	□No change (0)	□Higher (+1)

8. If feeling hot outdoors and unable to go indoors, what improvement measures would you take? [Multiple choices] *

□Move to shaded area	□Move to windy area	□Reduce activity	□Reduce clothing	□Use sunshade/umbrella or apply sunscreen	□Drink cold drinks

9. What do you think are the main factors affecting your outdoor activity in this space? [Multiple choices] * (Select at least 1 and up to 7 options)

□Weather is nice	□Distance to dorm/study/ workplace	□Whether vegetation is rich	□Whether facilities are complete	□Whether space is open	□Whether activity site is quiet	□Whether activity site is hygienic

### Appendix A.2

Winter Thermal Comfort Survey for Outdoor Leisure Squares on University Campuses

Note: This section is to be completed by the investigator and must be filled out at the time of the interview.

Survey Date	___ December 2024
Time of Completion	: - :

Time period of the interview [Single choice]*:

□9:00–10:00	□10:00–11:00	□11:00–12:00	□12:00–13:00
□13:00–14:00	□14:00–15:00	□15:00–16:00	□16:00–17:00 □17:00–18:00

Mark your current location

(Please use an “X” on the image to indicate your specific location within the plaza):

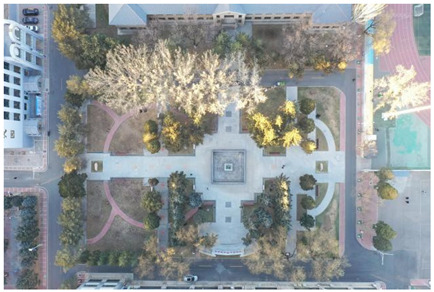

Area you are currently in during the interview [Single choice] *:

□Open sunny area □Light–shade interface □Building shaded area □Tree-shaded area

Part I: Personal Basic Information Survey (Respondents may answer this section repeatedly, but each response must be at least 30 min apart)

1. Gender: □ Male□ Female;

2. Age: _______________(years);

3. Height: __________(m);

4. Weight: _______________(kg)

5. Clothing—Upper body [Multiple choices depending on situation] *:

□Thermal underwear/pajamas (0.20 clo)	□Thin undershirt (short sleeves) (0.13 clo)	□Thick sleeveless vest (0.22 clo)	□Light long-sleeve sweater (0.25 clo)	□Heavy long-sleeve sweater (0.36 clo)
□Sweatshirt (0.30 clo)	□Light jacket (0.36 clo)	□Thick jacket (0.44 clo)	□Padded cotton coat (0.50 clo)	□Down jacket (0.55 clo)

6. Clothing—Lower body [Multiple choices depending on situation] *:

□Thermal long johns/pajama pants (0.15 clo)	□Thin wool trousers (0.24 clo)	□Thick wool trousers (0.28 clo)	□Thin long trousers (0.24 clo)	□Thick long trousers/fleece-lined pants (0.28 clo)

7. Footwear [Multiple choices depending on situation] *:

□Ankle-length athletic socks (0.02 clo)	□Stockings (0.02 clo)	□Calf-length socks (0.03 clo)	□Thick knee socks (0.06 clo)
□Seasonal shoes (winter/spring) (0.10 clo)	□Boots (0.10 clo)	□Leather shoes (0.04 clo)	□Cotton slippers (0.03 clo)

8. Your primary outdoor activity [Single choice] *:

□ Sitting (e.g., sunbathing, using phone, reading, chatting) (60W) (M < 1.2 met) □ Standing (e.g., reading info boards, light talking) (70W) (M < 1.2 met) □ Low-intensity exercise (e.g., walking < 1.2 m/s, Tai Chi, slow dancing) (150W) (1.2 ≤ M < 2.6 met)
□ Moderate-intensity exercise (e.g., brisk walking, fitness, dancing) (220W) (2.6 ≤ M < 3.8 met) □ High-intensity exercise (e.g., running, ball games) (360W) (M > 3.8 met)

Part II—Outdoor Space Use and Perception Survey

1. How long have you lived here? [Single choice] *:

□<6 months	□6 months–1 year	□1–3 years	□More than 3 years

2. On weekends/holidays, when do you most often stay in this space? [Multiple choices] *: (Note: Select at least 1 and up to 10 options)

□8:00–9:00	□9:00–10:00	□10:00–11:00	□11:00–12:00	□12:00–13:00
□13:00–14:00	□14:00–15:00	□15:00–16:00	□16:00–17:00	□17:00–18:00

3. What activity were you mainly doing 15 min ago? [Single choice] *:

□Lying down □Sitting □Low-intensity exercise (walking, Tai Chi, yoga, etc.) □Moderate-intensity exercise (brisk walking, jogging, etc.) □High-intensity exercise (ball games, competitive sports, etc.)

4. What kind of environment were you in 15 min ago? [Single choice] *:

□Indoors with air conditioning □Naturally ventilated indoors □Shaded outdoor space □Sunny outdoor space

5. What is your purpose here? [Single choice] *:

□Rest □Exercising □Socializing □Enjoy scenery □Reading □Group activity □Passing by

6. Frequency of outdoor activities in this space [Single choice] * (Note: More than once counts as multiple times):

□Daily □Several times a week □Weekly □Occasionally □Rarely

7. Duration of each outdoor visit [Single choice]*:

□<5min □5–15min □15–30min □>30min

8. Where do you mainly stay in the space? [Multiple choices]*

□Open sunny area □Tree-shaded area/building shade □Seating/rest area □Windless area □Constantly moving (if selected, no need to choose others; otherwise, select at least 1 and up to 3 options)

Part III—Outdoor Perception and Preferences Survey

1. How do you feel about the current thermal environment? [Single choice] *:

□Very cold (−3)	□Cold (−2)	□Cool (−1)	□Neutral (0)	□Slightly warm (+1)	□Warm (+2)	□Very hot (+3)

2. How comfortable do you feel now? [Single choice] *:

□Comfortable (0)	□Slightly uncomfortable(+1)	□Uncomfortable (+2)	□Very uncomfortable (+3)

3. To what extent do you accept the current thermal environment? [Single choice] *

□Fully acceptable (0)	□Slightly acceptable(−1)	□Slightly unacceptable(−1)	□Very unacceptable (−2)

4. Regarding current air temperature, you prefer: [Single choice] *

□Lower (−1)	□No change (0)	□Higher (+1)

5. Regarding current solar radiation, you prefer: [Single choice] *

□Lower (−1)	□No change (0)	□Higher (+1)

6. Regarding current wind speed, you prefer: [Single choice] *

□Lower (−1)	□No change (0)	□Higher (+1)

7. Regarding current relative humidity, you prefer: [Single choice] *

□Lower (−1)	□No change (0)	□Higher (+1)

8. If you feel cold outdoors and cannot go indoors, which measures would you mainly take to improve comfort? [Multiple choice]

□Move to a sunny area	□Move to a wind-sheltered area	□Increase activity intensity	□Add extra clothing	□Wear cold-weather gear (hat, mask, gloves)	□Drink a hot beverage (including plain hot water)

9. What do you think are the main factors affecting your outdoor activity in this space? [Multiple choices] * (Select at least 1 and up to 7 options)

□Weather is nice	□Distance to dorm/study/ workplace	□Whether vegetation is rich	□Whether facilities are complete	□Whether space is open	□Whether activity site is quiet	□Whether activity site is hygienic

### Appendix A.3

**Table A1:** L16(4^5^) orthogonal table and corresponding cases.

	Factor	Greening Layouts (A)	Plant Types (B)	Surface Albedo (C)	-	Error	Research Cases
Test Number							
1		1	1	1	1	1	
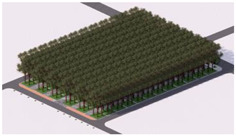
2		1	2	2	2	2	
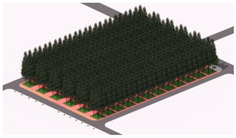
3		1	3	3	3	3	
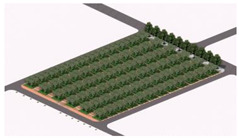
4		1	4	4	4	4	
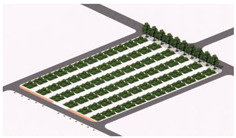
5		2	1	2	3	4	
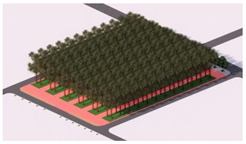
6		2	2	1	4	3	
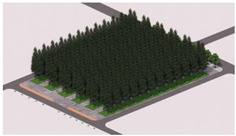
7		2	3	4	1	2	
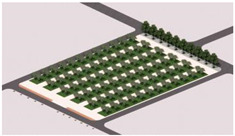
8		2	4	3	2	1	
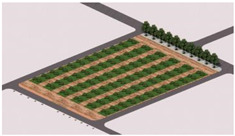
9		3	1	3	4	2	
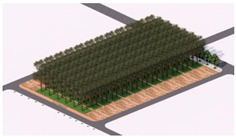
10		3	2	4	3	1	
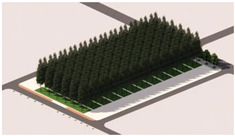
11		3	3	1	2	4	
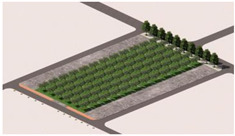
12		3	4	2	1	3	
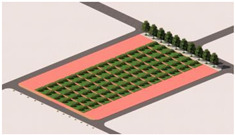
13		4	1	4	2	3	
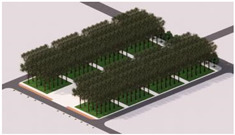
14		4	2	3	1	4	
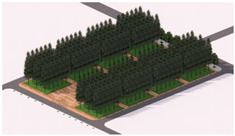
15		4	3	2	4	1	
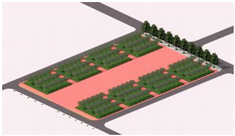
16		4	4	1	3	2	
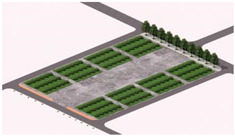

### Appendix A.4

During the summer period from 13:00 to 14:00, the calculation steps for the ($R_{BL}$) of the plant types (B) are as follows:

(A1) $${PET}_{B1}={PET}_{Case1}+{PET}_{Case5}+{PET}_{Case9}+{PET}_{Case13}$$

(A2) $${PET}_{B2}={PET}_{Case2}+{PET}_{Case6}+{PET}_{Case10}+{PET}_{Case14}$$

(A3) $${PET}_{B1}={PET}_{Case3}+{PET}_{Case7}+{PET}_{Case11}+{PET}_{Case15}$$

(A4) $${PET}_{B1}={PET}_{Case4}+{PET}_{Case8}+{PET}_{Case12}+{PET}_{Case16}$$

(A5) $$\bar{{PET}_{B1}}=\frac{{PET}_{B1}}{4};\;\bar{{PET}_{B2}}=\frac{{PET}_{B2}}{4};\;\bar{{PET}_{B3}}=\frac{{PET}_{B3}}{4};\;\bar{{PET}_{B4}}=\frac{PETB4}{4}$$

(A6) $$R_{BL}=max(\bar{{PET}_{BL}})-min(\bar{{PET}_{BL}})$$

### Appendix A.5

During the summer period from 13:00 to 14:00, the calculation steps for assessing the significance of the plant types (B) on the PET are as follows:

(A7) $${SS}_{B}=\frac{\sum_{i=1}^{4}{{PET}_{BL}}^{2}}{n}-\frac{{\left(\sum_{i=1}^{16}PET(Casei)\right)}^{2}}{m}$$

(A8) $${df}_{B}=n-1\;\;\;\;\;$$

(A9) $${df}_{total}=m-1$$

(A10) $${{df}_{error}=df}_{total}-\sum_{i=1}^{4}{df}_{factori}$$

(A11) $${MS}_{B}=\frac{{SS}_{BL}}{{df}_{B}}$$

(A12) $$F_{B}=\frac{{MS}_{B}}{{MS}_{error}}$$

(A13) $$Ρ=1-F_{CDF}(F_{B},{df}_{B},{df}_{error})\;\;$$

In these formulas:

- n: number of factor levels, n = 4;
- m: total number of cases, m = 16;
- ${SS}_{B}$: sum of squares for factor B;
- ${df}_{B}$: degrees of freedom for factor B;
- ${df}_{total}$: total degrees of freedom;
- ${df}_{error}$: degrees of freedom for error;
- ${MS}_{B}$: mean square for factor B;
- ${MS}_{error}$: mean square for error;
- $F_{B}$: F-value used to test whether factor B has a significant effect on the PET. A larger F-value indicates a more significant influence of the factor on the experimental results;
- $F_{CDF}$: the cumulative distribution function value of the F-distribution;
- Ρ: the significance level of the result. If the *p*-value is less than the predetermined significance level (e.g., Ρ < 0.05), it indicates that the factor has a significant impact on the result.

### Appendix A.6

The calculation steps for the contribution rate of the landscape elements to the PET during different activity periods in both summer and winter are as follows:

(A14) $$\rho_{F}=\frac{{SS}_{F}}{\sum {SS}_{F}}$$

(A15) $$\rho_{W,F}=0.5\rho_{W,F}^{\left(1\right)}+0.5\rho_{W,F}^{\left(2\right)}$$

(A16) $$\rho_{S,F}=0.5\rho_{S,F}^{\left(1\right)}+0.5\rho_{S,F}^{\left(2\right)}$$

(A17) $$\rho_{F}=0.82\rho_{W,F}+0.18\rho_{S,F}$$

In these formulas:

- ${SS}_{F}$: the sum of squares for the F-th factor, where F = A, B, C, and similarly for other factors;
- $\sum {SS}_{F}$: sum of squares for all factors;
- $\rho_{F}$: the contribution rate of the F-th factor to the PET variation for that time period;
- $\rho_{W,F}^{\left(1\right)}$: the contribution rate of the F-th factor to the PET variation during the first winter period (11:00–12:00), where the coefficient 0.5 represents the time period weighting;
- $\rho_{W,F}^{\left(2\right)}$: the contribution rate of the F-th factor to the PET variation during the second winter period (16:00–17:00), where the coefficient 0.5 represents the time period weighting;
- $\rho_{S,F}^{\left(1\right)}$: the contribution rate of the F-th factor to the PET variation during the first summer period (13:00–14:00), where the coefficient 0.5 represents the time period weighting;
- $\rho_{S,F}^{\left(2\right)}$: the contribution rate of the F-th factor to the PET variation during the second summer period (17:00–18:00), where the coefficient 0.5 represents the time period weighting;
- $\rho_{W,F}$: the weighted value of the contribution rate of the F-th factor to the PET variation during the main winter activity periods (11:00–12:00 and 16:00–17:00), where the coefficient 0.82 represents the winter weighting;
- $\rho_{S,F}$: the weighted value of the contribution rate of the F-th factor to the PET variation during the main summer activity periods (13:00–14:00 and 17:00–18:00), where the coefficient 0.18 represents the summer weighting;
- $\rho_{F}$: the combined weighted value of the contribution rate of the F-th factor to the PET variation during the main activity periods in both summer and winter (11:00–12:00 and 16:00–17:00 and 13:00–14:00 and 17:00–18:00).

### Appendix A.7

The calculation steps for selecting the optimal experimental scheme for thermal comfort in both summer and winter:

(A18) $$R_{w,i}=0.5r_{w,i}^{\left(1\right)}+0.5r_{w,i}^{\left(2\right)}$$

(A19) $$R_{s,i}=0.5r_{s,i}^{\left(1\right)}+0.5r_{s,i}^{\left(2\right)}$$

(A20) $$R_{i}=0.82R_{w,i}+0.18R_{s,i}$$

(A21) $$∆R=R_{i}-R_{O}$$

In these formulas:

- $r_{w,i}^{\left(1\right)}$: the percentage of the activity area within the neutral PET range to the total site area for scheme i during the first winter period (11:00–12:00), with a time period weighting coefficient of 0.5;
- $r_{w,i}^{\left(2\right)}$: the percentage of the activity area within the neutral PET range to the total site area for scheme i during the second winter period (16:00–17:00), with a time period weighting coefficient of 0.5;
- $r_{s,i}^{\left(1\right)}$: the percentage of the activity area within the neutral PET range to the total site area for scheme i during the first summer period (13:00–14:00), with a time period weighting coefficient of 0.5;
- $r_{s,i}^{\left(2\right)}$: the percentage of the activity area within the neutral PET range to the total site area for scheme i during the second summer period (17:00–18:00), with a time period weighting coefficient of 0.5;
- $R_{w,i}$: the weighted value of the percentage of the activity area within the neutral PET range to the total site area for scheme i during the main winter activity periods (11:00–12:00 and 16:00–17:00), with a winter weighting coefficient of 0.82;
- $R_{s,i}$: the weighted value of the percentage of the activity area within the neutral PET range to the total site area for scheme i during the main summer activity periods (13:00–14:00 and 17:00–18:00), with a summer weighting coefficient of 0.18;
- $R_{i}$: the comprehensive weighted value of the percentage of the activity area within the neutral PET range to the total site area for scheme i during the main activity periods in both summer and winter (11:00–12:00, 16:00–17:00, 13:00–14:00, and 17:00–18:00);
- $R_{O}$: the comprehensive weighted value of the percentage of the activity area within the neutral PET range to the total site area for the original scheme during the main activity periods in both summer and winter (11:00–12:00, 16:00–17:00, 13:00–14:00, and 17:00–18:00);
- ∆R: the comprehensive weighted improvement value of the percentage of the activity area within the neutral PET range for each experimental scheme compared to the original scheme during the main activity periods in both summer and winter.

### Appendix A.8

The PET distribution maps before and after the plaza renovation in summer and winter

## References

[1] Pörtner H.O., Roberts D.C., Tignor M., Poloczanska E.S., Mintenbeck K., Alegría A.C.M., Langsdorf S., Löschke S., Möller V., Contribution of Working Group II to the Sixth Assessment Report of the Intergovernmental Panel on Climate Change (2022). IPCC, 2022: Climate Change 2022: Impacts, Adaptation and Vulnerability.

[2] Fei F., Wang Y., Wang L., Fukuda H., Yao W. (2023). Influence of greenery configuration on summer thermal environment of outdoor recreational space in elderly care centers. Build. Environ..

[3] Lai D., Liu W., Gan T., Liu K., Chen Q. (2019). A review of mitigating strategies to improve the thermal environment and thermal comfort in urban outdoor spaces. Sci. Total Environ..

[4] Canan F., Golasi I., Falasca S., Salata F. (2020). Outdoor thermal perception and comfort conditions in the Koppen-Geiger climate category BSk. One-year field survey and measurement campaign in Konya, Turkey. Sci. Total Environ..

[5] Bian G.M., Gao X.Y., Zou Q.S., Cheng Q., Sun T.Y., Sha S.Y., Zhen M. (2023). Effects of thermal environment and air quality on outdoor thermal comfort in urban parks of Tianjin, China. Environ. Sci. Pollut. Res..

[6] Dashti A., Mohammadsharifi N., Shokuhi M., Matzarakis A. (2024). A comprehensive study on wintertime outdoor thermal comfort of blue-green infrastructure in an arid climate: A case of Isfahan, Iran. Sustain. Cities Soc..

[7] Heidari A., Davtalab J., Sargazi M.A. (2024). Effect of awning on thermal comfort adjustment in open urban space using PET and UTCI indexes: A case study of Sistan region in Iran. Sustain. Cities Soc..

[8] He X.Y., An L., Hong B., Huang B.Z., Cui X. (2020). Cross-cultural differences in thermal comfort in campus open spaces: A longitudinal field survey in China’s cold region. Build. Environ..

[9] Niu J.Q., Hong B., Geng Y.B., Mi J.Y., He J.Y. (2020). Summertime physiological and thermal responses among activity levels in campus outdoor spaces in a humid subtropical city. Sci. Total Environ..

[10] Geng Y.B., Hong B., Du M., Yuan T.T., Wang Y.B. (2022). Combined effects of visual-acoustic-thermal comfort in campus open spaces: A pilot study in China’s cold region. Build. Environ..

[11] Li Y.J., Hong B., Wang Y.B., Bai H.F., Chen H.Y. (2022). Assessing heat stress relief measures to enhance outdoor thermal comfort: A field study in China’s cold region. Sustain. Cities Soc..

[12] Chang J.Y., Du M., Hong B., Qu H.Y., Chen H.Y. (2023). Effects of thermal-olfactory interactions on emotional changes in urban outdoor environments. Build. Environ..

[13] Jing W.Q., Qin Z.M., Mu T., Ge Z.M., Dong Y.T. (2024). Evaluating thermal comfort indices for outdoor spaces on a university campus. Sci. Rep..

[14] Xiong J., Cheng B., Zhang J., Liu Y.S., Tan X.Y., Shi M.J., He X.M., Guo J.R. (2023). A study of waterside microenvironmental factors and their effects on summer outdoor thermal comfort in a Cfa-climate campus. J. Therm. Biol..

[15] Zhang Y.C., Liu J.L., Zheng Z.M., Fang Z.S., Zhang X.L., Gao Y.F., Xie Y.X. (2020). Analysis of thermal comfort during movement in a semi-open transition space. Energy Build..

[16] Lam C.K.C., Weng J.F., Liu K., Hang J. (2023). The effects of shading devices on outdoor thermal and visual comfort in Southern China during summer. Build. Environ..

[17] Yang J.H., Li H.Y., Fang Z.S., Li Y.C., Lu F.Q., Guo T.Y., Zhang X., Lin C., Lu J. (2025). Study on thermal and physiological responses during summer while moving between academic buildings under different walking conditions. Case Stud. Therm. Eng..

[18] Li J.N., Niu J.L., Huang T.Y., Mak C.M. (2022). Dynamic effects of frequent step changes in outdoor microclimate environments on thermal sensation and dissatisfaction of pedestrian during summer. Sustain. Cities Soc..

[19] Al Tahir I., El Fattah A.A., Mohammed M., Asif M., Almahdy O. (2025). Evaluating the performance of outdoor shading devices on human thermal comfort in hot humid climates: A case study of Dhahran. Build. Environ..

[20] Aghamohammadi N., Fong C.S., Idrus M.H.M., Ramakreshnan L., Haque U. (2021). Outdoor thermal comfort and somatic symptoms among students in a tropical city. Sustain. Cities Soc..

[21] Othman N.E., Zaki S.A., Rijal H.B., Ahmad N.H., Abd Razak A. (2021). Field study of pedestrians’ comfort temperatures under outdoor and semi-outdoor conditions in Malaysian university campuses. Int. J. Biometeorol..

[22] Tochaiwat K., Rinchumphu D., Sundaranaga C., Pomsurin N., Chaichana C., Khuwuthyakorn P., Phichetkunbodee N., Chan Y.C. (2023). The potential of a tree to increase comfort hours in campus public space design. Energy Rep..

[23] Khalili S., Fayaz R., Zolfaghari S.A. (2022). Analyzing outdoor thermal comfort conditions in a university campus in hot-arid climate: A case study in Birjand, Iran. Urban Clim..

[24] Andreou E. (2013). Thermal comfort in outdoor spaces and urban canyon microclimate. Renew. Energy.

[25] Cao X., Onishi A., Chen J., Imura H. (2010). Quantifying the cool island intensity of urban parks using ASTER and IKONOS data. Landsc. Urban Plan..

[26] Li Y., Ren C., Ho J.Y.-e., Shi Y. (2023). Landscape metrics in assessing how the configuration of urban green spaces affects their cooling effect: A systematic review of empirical studies. Landsc. Urban Plan..

[27] Zhou W., Huang G., Cadenasso M.L. (2011). Does spatial configuration matter? Understanding the effects of land cover pattern on land surface temperature in urban landscapes. Landsc. Urban Plan..

[28] Altunkasa C., Uslu C. (2020). Use of outdoor microclimate simulation maps for a planting design to improve thermal comfort. Sustain. Cities Soc..

[29] Dong Q., Xu X., Zhen M. (2023). Assessing the cooling and buildings’ energy-saving potential of urban trees in severe cold region of China during summer. Build. Environ..

[30] Taha H. (1997). Urban climates and heat islands: Albedo, evapotranspiration, and anthropogenic heat. Energy Build..

[31] Santamouris M., Gaitani N., Spanou A., Saliari M., Giannopoulou K., Vasilakopoulou K., Kardomateas T. (2012). Using cool paving materials to improve microclimate of urban areas–Design realization and results of the flisvos project. Build. Environ..

[32] Taleghani M., Berardi U. (2018). The effect of pavement characteristics on pedestrians’ thermal comfort in Toronto. Urban Clim..

[33] Rosso F., Pisello A.L., Cotana F., Ferrero M. (2016). On the thermal and visual pedestrians’ perception about cool natural stones for urban paving: A field survey in summer conditions. Build. Environ..

[34] Yue W., Liu X., Zhou Y., Liu Y. (2019). Impacts of urban configuration on urban heat island: An empirical study in China mega-cities. Sci. Total Environ..

[35] Karimi A., Sanaieian H., Farhadi H., Norouzian-Maleki S. (2020). Evaluation of the thermal indices and thermal comfort improvement by different vegetation species and materials in a medium-sized urban park. Energy Rep..

[36] Gachkar D., Taghvaei S.H., Norouzian-Maleki S. (2021). Outdoor thermal comfort enhancement using various vegetation species and materials (case study: Delgosha Garden, Iran). Sustain. Cities Soc..

[37] Chu Z.T., Li S., Li T., Qian H.J., Liu C., Yan Z.H. (2024). Numerical simulation of layout and landscape elements on the thermal environment of urban squares. Ecol. Inform..

[38] Zhu K., Huang B., Guo J. (1984). Natural Geography of China.

[39] Ministry of Housing and Urban-Rural Development of the People’s Republic of China (2016). Code for Thermal Design of Civil Buildings.

[40] NASA POWER. Data Access Viewer— Prediction of Worldwide Energy Resource. https://power.larc.nasa.gov/data-access-viewer/.

[41] (2019). Climate Season Classification.

[42] China Meteorological Administration Climate, Information Center Meteorological Data Office, Department of Building Science, Tsinghua University (2005). Standard Weather Data for Thermal Design of Buildings in China.

[43] Hall I., Prairie R., Anderson H., Boes E. Generation de typical meteorologicalvears for 26 SOLET stations. Proceedings of the Annual Meeting of the American Section of the International Solar Energy Society.

[44] Yang L., Lam J.C., Liu J. (2007). Analysis of typical meteorological years in different climates of China. Energy Convers. Manag..

[45] (1998). Ergonomics of the Thermal Environment-Instruments for Measuring Physical Quantities.

[46] Chow W.T.L., Akbar S., Heng S.L., Roth M. (2016). Assessment of measured and perceived microclimates within a tropical urban forest. Urban For. Urban Green..

[47] Spagnolo J., De Dear R. (2003). A field study of thermal comfort in outdoor and semi-outdoor environments in subtropical Sydney Australia. Build. Environ..

[48] Du H., Lian Z.W., Lan L., Lai D.Y. (2023). Application of statistical analysis of sample size: How many occupant responses are required for an indoor environmental quality (IEQ) field study. Build. Simul..

[49] Wang Z., Zhang H., He Y.D., Luo M.H., Li Z.W., Hong T.Z., Lin B.R. (2020). Revisiting individual and group differences in thermal comfort based on ASHRAE database. Energy Build..

[50] Guo T.Y., Lin Z.X., Zhao Y., Fang Z.S., Fan Y.N., Zhang X., Yang J.H., Li Y.K. (2024). Investigation and optimization of outdoor thermal comfort in elementary school campuses: Example from a humid-hot area in China. Build. Environ..

[51] Cronbach L.J. (1951). Coefficient alpha and the internal structure of tests. Psychometrika.

[52] Gagge A.P. (1971). An effective temperature scale based on a simple model of human physiological regulatory response. ASHRAE Trans..

[53] Höppe P. (1999). The physiological equivalent temperature—A universal index for the biometeorological assessment of the thermal environment. Int. J. Biometeorol..

[54] Tao Z.Y., Zhu X.D., Xu G.Q., Zou D.Z., Li G. (2023). A Comparative Analysis of Outdoor Thermal Comfort Indicators Applied in China and Other Countries. Sustainability.

[55] Matzarakis A., Rutz F., Mayer H. (2007). Modelling radiation fluxes in simple and complex environments—Application of the RayMan model. Int. J. Biometeorol..

[56] Sodoudi S., Zhang H.W., Chi X.L., Müller F., Li H.D. (2018). The influence of spatial configuration of green areas on microclimate and thermal comfort. Urban For. Urban Green..

[57] McPherson E.G., Nowak D.J., Rowntree R.A. (1994). Chicago’s Urban Forest Ecosystem: Results of the Chicago Urban Forest Climate Project.

[58] Cox D.R., Reid N. (2000). The Theory of the Design of Experiments.

[59] Condra L. (2001). Reliability Improvement with Design of Experiment.

[60] Chen L.X., Zhang Y.Z., Luo Z.Z., Yao F. (2022). Optimization Design of the Landscape Elements in the Lhasa Residential Area Driven by an Orthogonal Experiment and a Numerical Simulation. Int. J. Environ. Res. Public Health.

[61] Yang S.H., Zhou D., Wang Y.P., Li P. (2020). Comparing impact of multi-factor planning layouts in residential areas on summer thermal comfort based on orthogonal design of experiments (ODOE). Build. Environ..

[62] Zheng G.R., Xu H., Liu F., Dong J.W. (2024). Impact of Plant Layout on Microclimate of Summer Courtyard Space Based on Orthogonal Experimental Design. Sustainability.

[63] Yang Y.J., Gatto E., Gao Z., Buccolieri R., Morakinyo T.E., Lan H.N. (2021). The “plant evaluation model” for the assessment of the impact of vegetation on outdoor microclimate in the urban environment. Sustain. Cities Soc..

[64] Lam C.K.C., Lee H., Yang S.R., Park S. (2021). A review on the significance and perspective of the numerical simulations of outdoor thermal environment. Sustain. Cities Soc..

[65] Tsoka S., Tsikaloudaki A., Theodosiou T. (2018). Analyzing the ENVI-met microclimate model’s performance and assessing cool materials and urban vegetation applications-A review. Sustain. Cities Soc..

[66] Tominaga Y., Mochida A., Murakami S., Sawaki S. (2008). Comparison of various revised k–ε models and LES applied to flow around a high-rise building model with 1:1:2 shape placed within the surface boundary layer. J. Wind. Eng. Ind. Aerodyn..

[67] Forouzandeh A. (2018). Numerical modeling validation for the microclimate thermal condition of semi-closed courtyard spaces between buildings. Sustain. Cities Soc..

[68] Salata F., Golasi L., Volloaro R.D., Vollaro A.D. (2016). Urban microclimate and outdoor thermal comfort. A proper procedure to fit ENVI-met simulation outputs to experimental data. Sustain. Cities Soc..

[69] Stull R.B. (1988). An Introduction to Boundary Layer Meteorology.

[70] European Space Agency (ESA), German Aerospace Center (DLR) Sentinel-5P TROPOMI Cloud 1-Orbit L2 5.5km x 3.5km. https://disc.gsfc.nasa.gov/datasets/S5P_L2__CLOUD__HiR_2/summary?keywords=cloud%20cover.

[71] Cheung P.K., Jim C.Y. (2017). Determination and application of outdoor thermal benchmarks. Build. Environ..

[72] Yang Y.J., Zhou D., Wang Y.P., Meng X.Z., Gu Z.L., Xu D., Han X.X. (2022). Planning method of centralized greening in high-rise residential blocks based on improvement of thermal comfort in summer. Sustain. Cities Soc..

[73] Sun S.B., Xu X.Y., Lao Z.M., Liu W., Li Z.D., García E.H., He L., Zhu J.N. (2017). Evaluating the impact of urban green space and landscape design parameters on thermal comfort in hot summer by numerical simulation. Build. Environ..

[74] Zhang L., Zhan Q.M., Lan Y.L. (2018). Effects of the tree distribution and species on outdoor environment conditions in a hot summer and cold winter zone: A case study in Wuhan residential quarters. Build. Environ..

[75] Chen X., Xue P.N., Liu L., Gao L.X., Liu J. (2018). Outdoor thermal comfort and adaptation in severe cold area: A longitudinal survey in Harbin, China. Build. Environ..

[76] Su Y., Wang C.J., Li Z.M., Meng Q.L., Gong A., Wu Z.R., Zhao Q.F. (2024). Summer outdoor thermal comfort assessment in city squares-A case study of cold dry winter, hot summer climate zone. Sustain. Cities Soc..

[77] Canan F., Golasi I., Ciancio V., Coppi M., Salata F. (2019). Outdoor thermal comfort conditions during summer in a cold semi-arid climate. A transversal field survey in Central Anatolia (Turkey). Build. Environ..

[78] Hadianpour M., Mahdavinejad M., Bemanian M., Nasrollahi F. (2018). Seasonal differences of subjective thermal sensation and neutral temperature in an outdoor shaded space in Tehran, Iran. Sustain. Cities Soc..

[79] Yang B., Olofsson T., Nair G., Kabanshi A. (2017). Outdoor thermal comfort under subarctic climate of north Sweden—A pilot study in Umea. Sustain. Cities Soc..

[80] Mi J.Y., Hong B., Zhang T., Huang B.Z., Niu J.Q. (2020). Outdoor thermal benchmarks and their application to climate-responsive designs of residential open spaces in a cold region of China. Build. Environ..

[81] de Dear R.J., Brager G.S. (1997). Developing an Adaptive Model of Thermal Comfort and Preference.

[82] Yin Q., Cao Y.H., Sun C. (2021). Research on outdoor thermal comfort of high-density urban center in severe cold area. Build. Environ..

[83] Salata F., Golasi I., Vollaro R.D., Vollaro A.D. (2016). Outdoor thermal comfort in the Mediterranean area. A transversal study in Rome, Italy. Build. Environ..

[84] Nikolopoulou M., Steemers K. (2003). Thermal comfort and psychological adaptation as a guide for designing urban spaces. Energy Build..

[85] de Abreu-Harbicha L.V., Labakia L.C., Matzarakis A. (2015). Effect of tree planting design and tree species on human thermal comfort in the tropics. Landsc. Urban Plan..

[86] Zheng S.L., Guldmann J.M., Liu Z.X., Zhao L.H. (2018). Influence of trees on the outdoor thermal environment in subtropical areas: An experimental study in Guangzhou, China. Sustain. Cities Soc..

[87] Perini K., Chokhachian A., Auer T. (2018). Nature Based Strategies for Urban and Building Sustainability.

[88] Morakinyo T.E., Dahanayake K., Adegun O.B., Balogun A.A. (2016). Modelling the effect of tree-shading on summer indoor and outdoor thermal condition of two similar buildings in a Nigerian university. Energy Build..

[89] Taleghani M. (2018). The impact of increasing urban surface albedo on outdoor summer thermal comfort within a university campus. Urban Clim..

[90] Erell E., Pearlmutter D., Boneh D., Kutiel P.B. (2014). Effect of high-albedo materials on pedestrian heat stress in urban street canyons. Urban Clim..

[91] Wu Z.F., Chen L.D. (2017). Optimizing the spatial arrangement of trees in residential neighborhoods for better cooling effects: Integrating modeling with in-situ measurements. Landsc. Urban Plan..

[92] Abdi B., Hami A., Zarehaghi D. (2020). Impact of small-scale tree planting patterns on outdoor cooling and thermal comfort. Sustain. Cities Soc..

[93] Kong F.H., Yan W.J., Zheng G., Yin H.W., Cavan G., Zhan W.F., Zhang N., Cheng L. (2020). Retrieval of three-dimensional tree canopy and shade using terrestrial laser scanning (TLS) data to analyze the cooling effect of vegetation. Agric. For. Meteorol..

[94] Morakinyo T.E., Kong L., Lau K.K.L., Yuan C., Ng E. (2017). A study on the impact of shadow-cast and tree species on in-canyon and neighborhood’s thermal comfort. Build. Environ..

[95] Morakinyo T.E., Lam Y.F. (2016). Simulation study on the impact of tree-configuration, planting pattern and wind condition on street-canyon’s micro-climate and thermal comfort. Build. Environ..

[96] Morakinyo T.E., Lau K.K.L., Ren C., Ng E. (2018). Performance of Hong Kong’s common trees species for outdoor temperature regulation, thermal comfort and energy saving. Build. Environ..

[97] Guo W., Cheng B., Wang C.L., Tang X.Y. (2022). Tree planting indices and their effects on summer park thermal environment: A case study of a subtropical satellite city, China. Indoor Built Environ..

[98] Mballo S., Herpin S., Manteau M., Demotes-Mainard S., Bournet P.E. (2021). Impact of well-watered trees on the microclimate inside a canyon street scale model in outdoor environment. Urban Clim..

[99] Rahman M.A., Hartmann C., Moser-Reischl A., von Strachwitz M.F., Paeth H., Pretzsch H., Pauleit S., Rötzer T. (2020). Tree cooling effects and human thermal comfort under contrasting species and sites. Agric. For. Meteorol..

[100] Shi D.C., Song J.Y., Huang J.X., Zhuang C.Q., Guo R., Gao Y.F. (2020). Synergistic cooling effects (SCEs) of urban green-blue spaces on local thermal environment: A case study in Chongqing, China. Sustain. Cities Soc..

[101] Fei F., Wang L.Y., Wang Y., Yao W.X., Fukuda H., Xiao Y.L., Tian L., Ji T.T. (2023). A new method for evaluating the synergistic effect of urban water body and vegetation in the summer outdoor thermal environment. J. Clean. Prod..

[102] Liu Q.X., Dong Q., Zhang L.C., Sun C. (2024). Summer cooling island effects of blue-green spaces in severe cold regions: A case study of harbin, China. Build. Environ..

